# Harnessing machine learning for assessing climate change influences on groundwater resources: A comprehensive review

**DOI:** 10.1016/j.heliyon.2024.e37073

**Published:** 2024-08-28

**Authors:** Apoorva Bamal, Md Galal Uddin, Agnieszka I. Olbert

**Affiliations:** a School of Engineering, University of Galway, Galway, Ireland; b Ryan Institute, University of Galway, Galway, Ireland; c MaREI Research Centre, University of Galway, Galway, Ireland; d Eco-HydroInformatics Research Group (EHIRG), Civil Engineering, University of Galway, Galway, Ireland

**Keywords:** Climate change, Groundwater variables, Artificial intelligence, Machine learning, Ireland

## Abstract

Climate change is a major concern for a range of environmental issues including water resources especially groundwater. Recent studies have reported significant impact of various climatic factors such as change in temperature, precipitation, evapotranspiration, etc. on different groundwater variables. For this, a range of tools and techniques are widely used in the literature including advanced machine learning (ML) and artificial intelligence (AI) approaches. To the best of the authors’ knowledge, this review is one of the novel studies that offers an in-depth exploration of ML/AI models for evaluating climate change impact on groundwater variables. The study primarily focuses on the efficacy of various ML/AI models in forecasting critical groundwater parameters such as levels, discharge, storage, and quality under various climatic pressures like temperature and precipitation that influence these variables. A total of 65 research papers were selected for review from the year 2017–2023, providing an up-to-date exploration of the advancements in ML/AI methods for assessing the impact of climate change on various groundwater variables. It should be noted that the ML/AI model performance depends on the data attributes like data types, geospatial resolution, temporal scale etc. Moreover, depending on the research aim and objectives of the different studies along with the data availability, various sets of historical/observation data have been used in the reviewed studies Therefore, the reviewed studies considered these attributes for evaluating different ML/AI models. The results of the study highlight the exceptional ability of neural networks, random forest (RF), decision tree (DT), support vector machines (SVM) to perform exceptionally accurate in predicting water resource changes and identifying key determinants of groundwater level fluctuations. Additionally, the review emphasizes on the enhanced accuracy achieved through hybrid and ensemble ML approaches. In terms of Irish context, the study reveals significant climate change risks posing threats to groundwater quantity and quality along with limited research conducted in this avenue. Therefore, the findings of this review can be helpful for understanding the interplay between climate change and groundwater variables along with the details of the various tools and techniques including ML/AI approaches for assessing the impacts of climate changes on groundwater.

## Highlights

• Groundwater is vulnerable to climate-driven changes in weather patterns and hydrological processes.
• Neural networks, Random Forest, and Support Vector Machines are effective in forecasting critical groundwater parameters.
• Enhanced accuracy is achieved through hybrid and ensemble machine learning approaches.
• Preliminary correlation analysis reveals relationships between environmental parameter and groundwater varaibles.
• Data improvement strategies like interpolation and enhanced monitoring is required.

## Graphical abstract

**Figure:**
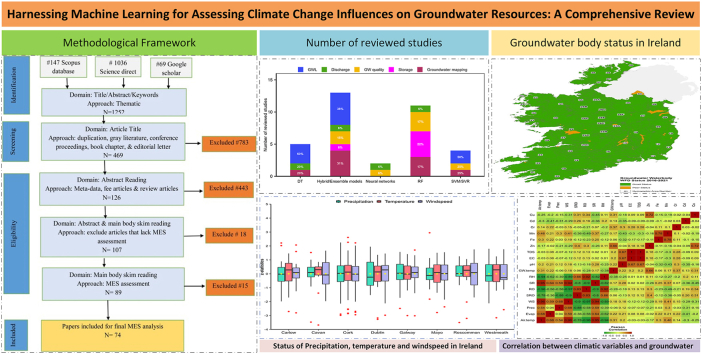

## 1 Introduction

Over the last 65 years, the world has experienced significant global changes driven by the observed and projected climatic shifts of the twenty-first century, commonly referred to as global warming [1]. This phenomenon gives rise to climate change, a multifaceted global challenge with far-reaching impacts on various facets of ecology, environment, socio-politics, and socio-economics, necessitating international collaboration [1,2]. Climate change is identified by examining long-term temperature and precipitation trends, as well as other factors like pressure and humidity in the surrounding environment [3]. Notable consequences of climate change encompass unpredictable weather patterns, the recession of global ice sheets, and the escalation of sea levels, bearing implications on both the international and domestic fronts [4,5]. Groundwater, a readily accessible and high-quality source of freshwater, exhibits a close relationship with surface water [6]. Approximately 85 % of groundwater extractions originate from surface water sources with reduced evapotranspiration, while the remaining 15 % stem from aquifer depletion [7]. In numerous environments, natural groundwater discharge plays a pivotal role in sustaining baseflow to rivers, lakes, and wetlands during periods of drought [[8], [9], [10]].

The impacts of climate change on hydrology and water management have extensive consequences, including the degradation of river and riparian ecosystems [[11], [12], [13]]. Therefore, it is imperative to develop a comprehensive understanding of the potential effects of anticipated climate changes on various aspects, including freshwater resource availability and reliability, water demand, flood and drought frequencies, as well as the preservation of existing animal and plant species and water-related infrastructure [[14], [15], [16]]. To achieve this understanding, it is vital to prioritize both national and international programs that encompass comprehensive monitoring, research, and control efforts. Water-related data play a fundamental role in conducting studies related to climate changes and variability [17,18]. Climate variability and change have direct effects on groundwater systems through processes like replenishment via recharge [19] and indirect effects through alterations in groundwater usage, influenced by human activities such as land-use changes [4,20].

To anticipate future groundwater risks, it is essential to consider a complex interplay of factors, including climate variations, land use, soil characteristics, landscape features, geological conditions, and hydrological systems [10,21,22]. Climate change also exerts its influence on critical variables like soil moisture and temperature, which have far-reaching implications for terrestrial ecosystems and water resources, ultimately affecting groundwater availability and flow dynamics [23]. The long-term responses of groundwater systems to climate shifts, independent of human activities, can be discerned through paleo-hydrological evidence, particularly in arid and semi-arid regions [6,9,[24], [25], [26], [27], [28]]. Groundwater replenishment processes encompass both diffuse rain-fed recharge and focused recharge from surface waters, contingent upon variables such as climate conditions, land cover, and geological characteristics [24,29]. Maintenance of groundwater levels is intricately linked to climate and land cover, governing precipitation and evapotranspiration rates [30,31]. Meanwhile, soil properties and geological features dictate the capacity to store excess water below the surface [32,33]. Furthermore, climate variability, including extreme events like droughts [34,35] and floods [36], yield considerable influence on groundwater recharge, potentially altering its duration and magnitude [37,38]. These dynamics of declining groundwater levels can exacerbate reduced summer stream flows, posing significant challenges for meeting diverse water needs, encompassing domestic, agricultural, and ecological requirements [[39], [40], [41]]. Additionally, preserving and restoring groundwater ecosystems, such as recharge areas, wetlands, and mountain forests, emerge as critical imperatives in safeguarding global water resources [42]. Understanding climate change's impact on groundwater variables requires precise and accurate monitoring methodologies [43]. Past studies emphasize the risk to social and environmental ecosystems due to disrupted groundwater security as a consequence of climate change [44,45]. To avoid underestimating future groundwater recharge and levels, daily groundwater-surface water coupled modelling is crucial, incorporating daily rainfall distribution [21]. However, due to extensive data requirements for such complex coupled models, our understanding of the mechanisms responsible for hydrological changes in groundwater-surface water interactions under climate change scenarios remains limited. Moreover, significant uncertainty surrounds groundwater residence times, including local and regional groundwater flows contributing to surface discharge. To address current and future groundwater challenges, focused research based on comprehensive meteorological and hydrological data is essential [14].

Over the years, various groundwater modelling methods have been employed, including numerical, physically based, statistical, and conceptual techniques. Statistical and multivariate statistical approaches have been valuable for assessing changes in groundwater variables, offering insights into trends and patterns related to groundwater level, storage, recharge, discharge, and quality [46,47]. Notably, MODFLOW (Modular Three-dimensional Finite-difference Ground-water Flow Model) stands out as a versatile computer program, seamlessly incorporating simulation capabilities and minimizing the need for extensive program alterations [48]. In supplementary material, Table S1 summarizes the advantages and challenges of various methods, including remote sensing-based techniques and hydrological and numerical models (e.g., SWAT, MIKE 11, WetSpass, Hydrus-1D, soil water balance models), utilized by researchers to assess climate change's impact on groundwater variables. While traditional approaches have some limitations, data-driven models excel in accurately forecasting concerned variables without delving into intricate underlying mechanisms [[49], [50], [51]].

Scientists have developed diverse methodologies to simulate groundwater variables, including levels, storage, discharge, and soil moisture, ranging from conceptual to numerical techniques, and incorporating artificial intelligence (AI) models such as machine learning (ML) [52]. ML, a subset of AI, focuses on creating algorithms that recognize patterns in data and make predictions [53]. Various ML models, like decision trees, random forests, support vector machines, and artificial neural networks, have gained prominence over the past decade to address the limitations of numerical methods in climate and water modeling [54]. ML models offer the advantage of conducting simulations without requiring in-depth knowledge of aquifer physical properties, making them highly appealing for such purposes [52,54,55]. Consequently, a range of ML models is employed to assess and predict groundwater variables concerning climate change and their effectiveness is compared. This comparison is conducted using various evaluation metrics, including accuracy, mean absolute error, root mean square error, mean square error, correlation, etc. [56,57]. Hence, ML/AI model with highest accuracy, and least error is chosen as the best model for assessing the impacts of climate change on groundwater variables. This comparative analysis provides valuable insights into each model's strengths and limitations, assisting researchers and practitioners in selecting the most suitable approach. For instance, ML/AI approaches are particularly valuable for understanding the inputs that most impact groundwater level predictions [58].

Similarly, groundwater storage and discharge have garnered attention for ML/AI deployment due to challenges associated with relying solely on remote sensing, including coarse resolution and spatial variation [59]. Changes in groundwater storage and agricultural pumping in active semi-arid regions are significant yet not comprehensively understood aspects of the water balance [59]. As surface-water storage dwindles, especially during droughts, the role of groundwater storage in mitigating drought effects becomes increasingly vital [34]. In the past, groundwater storage in aquifers was primarily influenced by local climate conditions, ecological requirements, and interactions with surface water [60]. However, during the latter half of the twentieth century, global declines in water-table levels and storage occurred due to widespread high-capacity well pump usage, human exploitation of aquifers, and a warming climate [61]. Groundwater potential mapping, aimed at assessing and predicting areas with scarce or abundant groundwater resources, shows great potential in harnessing ML/AI to enhance precision and effectiveness through diverse data inputs, advanced modelling techniques, and continuous real-time monitoring [62,63].

Additionally, most studies on climate change impact on groundwater have focused on processes like recharge, discharge [64], storage changes, and subsurface water flow mechanisms. Comparatively, fewer have examined climate change's effects on groundwater quality [22,65,66]. Groundwater quality depends on chemical, physical, and biological attributes, and it's expected to change due to climate shifts and human activities affecting recharge, discharge, and land use in groundwater systems [[67], [68], [69]]. Various tools and methods have been used in groundwater research to monitor quality, with the Water Quality Index (WQI) being widely adopted [[70], [71], [72], [73]]. WQI accurately assesses water quality using numeric scores and classifications, making it valuable for researchers [[73], [74], [75], [76], [77], [78], [79]]. For in-depth information on WQI models and applications, refer to Uddin et al. (2021). A recent study also improved monitoring programs and groundwater quality through hydrogeochemistry assessment using WQI [77,80].

In conclusion, this paper provides vital insights for hydrology and water resource management professionals by comparing machine learning models' assessment and prediction of groundwater variables. It represents a pioneering effort in reviewing ML/AI techniques that link climate change to multiple groundwater variables, offering comprehensive insights into recent research in this field.

The main objective of this review is to analyse the effects of climate change on groundwater variables using ML/AI and provide suggestions for conducting similar research in specific regions, such as Ireland, for better groundwater management scenarios. Moreover, the research aims to provide a thorough understanding of how different ML/AI methods have been used to interlink climate change with groundwater systems, impacting groundwater levels, discharge, storage, and overall quality and provide suggestions for conducting similar research in Ireland. The specific objectives of the paper are as follows.

• Literature review-thoroughly examine existing scientific literature, including studies, research papers, and reports, to gain insights into the utilization of ML/AI methods for assessing the impact of climate change on groundwater variables and explore the mechanisms by which climatic factors affect groundwater quality.
• Identify vital climate change drivers-identify and analyse various climatic drivers capable of influencing groundwater systems. This includes assessing alterations in precipitation patterns, temperature, evapotranspiration, and sea-level rise.
• Assessment of Ireland's climatic variables and groundwater condition- Assess Ireland's climate change attributes and groundwater information to acquire an in-depth understanding of the current climate change scenario in Ireland and proposes the potential of a framework for conducting analogous research in the region.
• Identify potential tools/techniques- Explore the potential avenue for utilizing ML/AI techniques in this field.

However, in pursuit of a holistic understanding, this research aims to elucidate the multifaceted applications of ML/AI methods in assessing the ramifications of climate change on groundwater resources, encompassing a wide spectrum of water quality and hydrological variables. Through a rigorous exploration of these intricate relationships, this study aspires to furnish invaluable insights that hold the potential to inform and enhance strategies for water resource management and adaptation, especially in the context of a dynamically changing climate.

## 2 Methodology

**Fig. 1:**
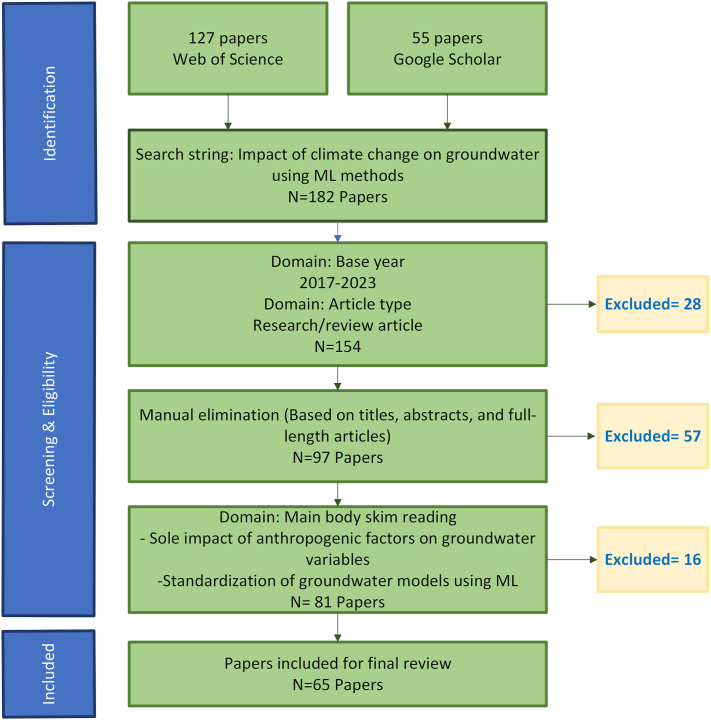
Methodological framework of the research.

The research employed a systematic literature review approach, with the methodological framework illustrated in Fig. 1. The methodology section is structured into the following sub-sections.

### 2.1 Comprehensive review of climate change impact on groundwater variables

Various academic databases, including Google Scholar, ResearchGate, Scopus, and Web of Science, were queried to identify pertinent studies. The selected studies underwent analysis based on modifications in groundwater variables resulting from climate change, methodologies for assessing this impact, and the relevance of data for planning adaptation strategies. These studies offer insights into the influence of climatic variables, either in isolation or in combination, on different groundwater variables.

### 2.2 Literature review of AI methods for assessing climate change and groundwater

An extensive search was conducted for articles in English, published in peer-reviewed journals since 2017, focusing on the assessment of climate change impacts on various groundwater variables. To ensure the comprehensiveness and rigor of the search, multiple iterations were performed, encompassing different search combinations. This approach aimed to provide a comprehensive overview of relevant papers, including those initially excluded due to variations in search terms and criteria (Fig. 1).

The search was conducted using 'Web of Science' and 'Google Scholar.' Initially, articles related to the "impact of climate change on groundwater using machine learning methods" were sought, yielding 182 papers. To ensure a focus on the most recent primary research utilizing machine learning, a base year of 2017 was introduced as a cutoff point, resulting in 154 publications published in total from the year 2017–2023. This choice aimed to review the latest research on the topic.

To refine the search, publications not aligned with the assessment of climate change's impact on groundwater using machine learning methods were manually eliminated. This involved a thorough examination of titles, abstracts, and, when necessary, full-length articles to assess the methodology's robustness and scientific relevance. The selected articles were required to be in English and feature abstracts presenting original research findings on the specified topic, with a primary emphasis on the application of machine learning methods for assessing climate change's impacts on groundwater variables. This process identified a total of 97 studies published in peer-reviewed journals, ensuring the research's quality and reliability.

Subsequently, the major exclusion criteria for this search were papers exclusively focusing on.

• The impact of anthropogenic factors on groundwater variables.
• Standardization of groundwater models using machine learning.

**Fig. 2:**
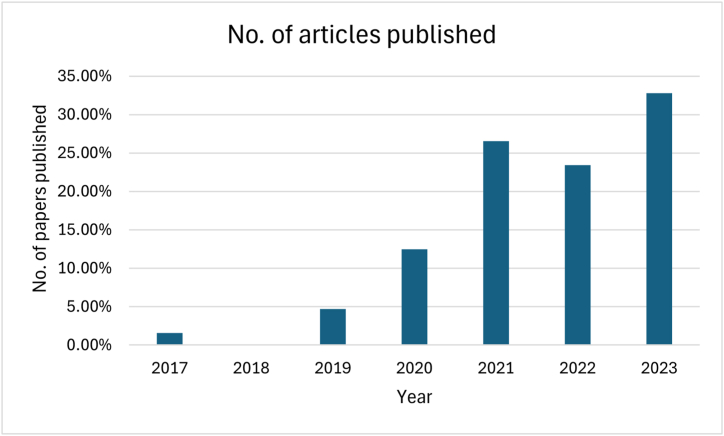
Number of articles published each year (2017–2023).

Following the application of these exclusion criteria, a total of 65 research papers were chosen for review, providing an up-to-date exploration of the advancements in machine learning methods for assessing the impact of climate change on various groundwater variables. Fig. 2 provides the details of the number of articles published each year, from 2017 to 2023.

## 3 Climate change and groundwater variables

Climate change significantly alters groundwater, influenced by natural climatic patterns and human-induced factors like global warming. These changes affect both the quality and quantity of groundwater, with varying impacts across different regions and over time [1,3,14,81].

### 3.1 Major climatic factors impacting groundwater variables

As climate change unfolds, it exerts a multifaceted influence on surface hydrology components, encompassing critical factors such as precipitation, temperature, evapotranspiration, extreme hydro-climatic events, sea-level rise, alterations in the hydrological cycle, shifts in carbon dioxide concentrations, changes in surface runoff, variations in stream flow, fluctuations in soil temperature, and adjustments in soil water content, as highlighted in studies by Ref. [82]. These perturbations ripple through the intricate web of hydrological processes and subsequently impact the subsurface hydrologic cycle, as noted by Ref. [83]. Given the broad spectrum of groundwater residence times, which can range from mere days to extensive years, the effects of climate change are dispersed both temporally and spatially. Consequently, detecting and comprehending the responses of groundwater hydrology to these myriad climate-driven changes has proven to be intricate challenges, as emphasized by previous research conducted by Refs. [84,85]. Despite these complexities, ongoing scientific efforts are dedicated to enhancing our understanding of these dynamic interactions, fostering more accurate predictions, and enabling proactive groundwater management strategies in the context of an evolving climate.

#### 3.1.1 Precipitation

Climate change has exerted a profound influence on precipitation patterns, both at regional and global scales, as substantiated by extensive research, including studies by Refs. [[86], [87], [88]]. These alterations in precipitation dynamics have brought about significant shifts in seasonal patterns, a phenomenon extensively investigated in works by Refs. [89,90]. The intricate interplay between precipitation and groundwater level fluctuations, specifically the impact of rainfall on groundwater dynamics, has been a subject of rigorous scientific inquiry. Researchers have developed sophisticated linear differential equations, as exemplified by the pioneering work of Ref. [91], shedding light on the quantifiable relationship between rainfall and groundwater level variations, a finding underscored by Ref. [92]. Furthermore, the scientific community has advanced analytical models that establish the nexus between groundwater levels and precipitation, with subsequent applications for estimating recharge in unconfined aquifers, as seen in studies conducted by Refs. [[93], [94], [95]]. Expanding on this knowledge [96], introduced innovative techniques, including weighted rainfall indices and statistical analyses like Pettitt and Mann–Kendall tests, elucidating that variations in groundwater levels are overwhelmingly governed by precipitation, solidifying its pivotal role in shaping aquifer system behavior. Further, Standardized Precipitation Index (SPI), the Standardized Precipitation Evapotranspiration Index (SPEI) and the Standardized Groundwater Index (SGI) have also been used to assess the impact of climate change on groundwater drought [97]. Departing from traditional methodologies, authors of Ref. [98]recently proposed a systematic approach for the comprehensive analysis of the temporal and frequency correlation between precipitation-related features and groundwater depth data. This novel method combines independent component analysis with wavelet coherence analysis, yielding deeper insights into the intricate response of groundwater dynamics to large-scale climatic patterns through precipitation. In addition to advancing our understanding, this approach provides a robust foundation for further research and result interpretation.

#### 3.1.2 Temperature

The persistence of groundwater dynamics in mountainous regions holds a substantial influence on the responses of streams to temperature changes, as elucidated by Ref. [99]. This observation gains further depth in the insights provided by Ref. [100], who underscore the pivotal role of groundwater dynamics, including processes like underground drainage, alongside topographical variations in snowy terrains, offering crucial insights into the anticipated responses of mountainous environments to climate fluctuations. Notably, authors of Ref. [101] employed temperature as the primary explanatory variable in their research, enabling the construction of scenarios to assess potential impacts of future temperature alterations on water table depths and salinity changes. This aligns with findings by Ref. [102], where temperature exerted a more pronounced influence on subsurface water levels in the study area compared to rainfall. The study attributed the more substantial decline in groundwater levels under the RCP8.5 scenario to the higher temperature projections associated with this scenario. Beyond its effects on groundwater depth and recharge rates, temperature has also emerged as a key determinant of groundwater quality, as highlighted by Ref. [22]. In a comprehensive study conducted in the USA, authors of Ref. [103] employed an integrated model encompassing both surface and groundwater systems. Their findings shed light on the sensitivity of shallow groundwater to warming, emphasizing that persistent warming can deplete storage capacity and disrupt vital hydrological connections. This underscores the profound and early ramifications that even minor to moderate temperature increases can exert on groundwater storage and evapotranspiration dynamics.

#### 3.1.3 Evapotranspiration

Like fluctuations in precipitation, climate change influences evapotranspiration rates through noticeable warming trends. Consequently, this leads to changes in soil water content and runoff dynamics [[104], [105], [106]]. It's important to note that precipitation and evapotranspiration are key factors in determining the rate of groundwater recharge [[107], [108], [109]]. Anticipated climate changes, including variations in precipitation, temperature, and evapotranspiration, are expected to have diverse and significant effects on different aquifers and specific areas within these aquifers [85,110,111]. These impacts will be closely linked with variations in hydraulic properties and proximity to recharge zones [8,24,112,113].

A study by the authors of Ref. [103] found that changes in subsurface water storage can support increased evapotranspiration as water demand rises. Notably, 85 % of the evapotranspiration response to warming occurred in the more humid eastern part of the study area. Additionally, recent research by the authors of Ref. [114] proposed a three-stage framework for examining the impact of climate on average annual groundwater evapotranspiration in west-central Florida. This study revealed that as the land gets drier, groundwater evaporation decreases. The study also showed that short-term climate changes impact groundwater evaporation the most, and areas with more forests and wetlands have higher groundwater evaporation compared to areas with more paved surfaces. Of the various climate variabilities affecting groundwater evapotranspiration, the most significant changes were caused by month-to-month climate variability, followed by year-to-year and daily climate variability.

#### 3.1.4 Extreme hydro-climatic events

The consequences of climate change manifest in various extreme hydro-climatic events that exert differential impacts on groundwater variables, contingent upon their frequency and magnitude. A significant analytical framework, as demonstrated by Ref. [115], was developed to evaluate the repercussions of compound inundation resulting from coastal climate change. This framework unravelled the intricacies of surface flow dynamics during extreme precipitation events while considering their interplay with rising groundwater levels and sea-level fluctuations in a coastal watershed proximate to Oakland. Complementing these findings, Ref. [116] recently assessed recurrent coastal flooding and its ramifications on groundwater dynamics, spotlighting an intriguing observation of heightened soil moisture content concurrent with increasing flood occurrences. This augmented moisture percolates into the groundwater segment of the aquifer. However, as annual flooding events continue to escalate, a pertinent study by Ref. [117] scrutinized the performance of the Soil and Water Assessment Tool (SWAT) model in groundwater-dominated regions, revealing its suboptimal performance compared to surface water regions.

In addition to influencing groundwater depth and recharge, extreme hydro-climatic events such as floods wield multifaceted effects on groundwater quality, as discerned in the comprehensive investigation by Ref. [118]. Their study, encompassing 26 samples, illuminated a nuanced pattern wherein 69 % of wells exhibited enhanced water quality post-flood conditions, while 19 % of well water samples experienced a deterioration in quality compared to pre-flood conditions. The remaining 12 % of samples displayed no significant alteration in quality during the flood period, encompassing diverse physical and chemical water quality parameters.

Extending beyond floods, a recent study conducted in Iran by the authors of Ref. [119] delves into the repercussions of drought on groundwater storage, unearthing a disconcerting trend of negative impacts. Under the RCP4.5 scenario, projections indicate an 8.8 % decline in groundwater storage compared to the base period of 1990–2016. This diminishment escalates to an estimated 9.5 % under the more severe RCP8.5 scenario, underscoring the dire implications of prolonged drought conditions on groundwater resources. Additionally, the study conducted by the authors of Ref. [120] analyzed the trends of the level of groundwater and groundwater drought using Standard Groundwater Level Index wherein it was reported that groundwater drought caused the reduction of groundwater recharge potential of the well.

#### 3.1.5 Sea level rise

The escalation in sea levels has led to an amplified incursion of saltwater into groundwater aquifers, posing a potential threat to the suitability of this water for human consumption due to heightened salinity levels [121,122]. Correspondingly, the authors of Ref. [123] conducted a study with the aim of characterizing the coastal aquifer on the North coast of Mombasa, employing a multifaceted approach. This approach encompassed the utilization of the GALDIT overlay index, groundwater quality index for evaluating seawater intrusion, measurement of total hardness, and application of the water quality index. Evaluation via the groundwater quality index unveiled that a predominant portion of the groundwater within the aquifer exhibits brackish characteristics, accounting for 68 % of the total volume. However, as one traverses towards the southern extremity of the aquifer, a transition to saline conditions is observed, constituting 32 % of the total groundwater.

Additionally, research by the authors of Ref. [124] underscores the consequences of a 1-m rise in sea level, with projections indicating that areas susceptible to inundation from below will extend approximately 50–130 m further inland. Notably, coastal topography plays a pivotal role in this phenomenon, as the progressive ascent of water tables over time intersects with low-lying drainage features. This intersecting dynamic facilitates groundwater discharge, consequently constraining the extent of sediment deposition in approximately 70 % of California's coastal water tables, with variations ranging from 68.9 % to 82.2 %. Failure to account for these topography-mediated responses results in a 20 % augmentation in flood-prone area estimations and substantial underestimations in saltwater intrusion. Regardless of the scenario, regions featuring shallow coastal water tables are anticipated to diminish in size due to inundation stemming from overland flooding or constraints imposed by expanding inland areas.

#### 3.1.6 Alterations in hydrological cycle

In the current epoch marked by transformative shifts in water resources, the global phenomenon of climate change is instigating rapid and profound alterations in the intricate system of the water cycle. These transformations materialize through a series of discernible manifestations, including the escalating frequency of extreme weather events, the dwindling cryosphere characterized by diminishing frozen water reserves, a persistent decline in groundwater levels, the degradation of aquatic ecosystems, and a heightened prevalence of water scarcity in various regions.

A notable study conducted by the authors of Ref. [125] sought to comprehensively assess regional groundwater recharge, employing version 3.07 of the Hydrological Evaluation of Landfill Performance (HELP) model. Their approach, grounded in meticulous daily water budget analysis, shed light on the dynamics of future groundwater recharge. The findings underscored that the projected changes in precipitation patterns held a dominant influence over estimated groundwater recharge, surpassing the significance of alterations in solar radiation and temperature patterns. Through extensive uncertainty analysis, it was unequivocally substantiated that variations in projected precipitation exerted a substantially more pronounced impact on potential recharge within the specific study area when juxtaposed with shifts in projected temperature.

In a subsequent investigation by the authors of Ref. [126], the study delved into the aftermath of Hurricane Maria, which triggered extensive deforestation in Puerto Rico. This catastrophic event unfolded in synchrony with an extended sequence of hydrological consequences that reverberated across multiple dimensions of the water cycle. These ramifications extended their reach into the domains of the atmosphere, terrestrial landscapes, and marine ecosystems, thereby underscoring the far-reaching and interconnected nature of climate-induced hydrological transformations.

### 3.2 Assessment of changes in groundwater variables using ML/AI techniques

In the reviewed literature, the primary emphasis has been on assessing the repercussions of various climatic changes on groundwater levels. These investigations have harnessed a diverse array of climatic parameters as input variables and employed various ML/AI techniques to elucidate the fluctuations in groundwater levels. However, the scope of ML/AI applications extends well beyond groundwater level assessments, encompassing a comprehensive array of other vital groundwater variables [37,43,127,[128], [129]]. Notably, ML/AI methods have been effectively utilized for the analysis of groundwater discharge, enabling insights into groundwater flow dynamics and discharge rate influencing factors [64,130,131]. Additionally, ML/AI algorithms have been instrumental in estimating changes in groundwater storage, aided by satellite-based remote sensing data [37,43]. Soil moisture monitoring, crucial for understanding groundwater recharge processes, has also benefited from ML-driven models [43]. Furthermore, ML/AI has been applied to assess groundwater quality changes, facilitating the identification of trends and anomalies in parameters influenced by climatic shifts [71,132]. Geospatial analyses powered by ML/AI have emerged as a powerful tool for mapping groundwater potential zones, integrating geological, topographical, and climatic variables [133,134]. Moreover, ML/AI approaches have contributed to our understanding of the intricate interactions between climatic variables and groundwater dynamics, shedding light on groundwater responses to changing climate conditions. These studies often adopt a multivariable approach, considering multiple groundwater parameters simultaneously, and employ uncertainty quantification techniques to address uncertainties associated with groundwater assessments [54,135,136]. In real-time monitoring and decision support systems, ML/ AI, coupled with IoT technology, enables continuous groundwater monitoring and proactive responses to be changing climatic conditions, bolstering sustainable groundwater resource management efforts. In sum, while most studies have cantered on groundwater level changes in response to climatic shifts, ML/AI techniques have broadened their horizons to encompass a wide spectrum of groundwater variables, enriching our ability to comprehensively analyse and manage groundwater resources amidst evolving climatic challenges.

**Fig. 3:**
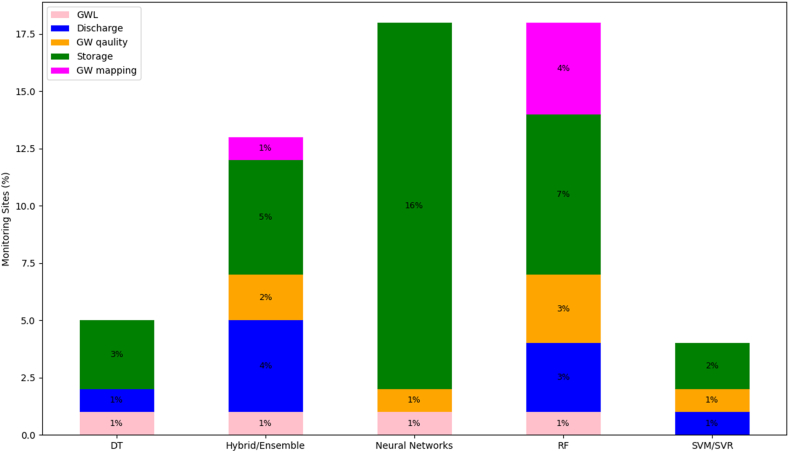
Number of reviewed studies using ML/AI for assessing groundwater variables.

However, the research identified a variety of algorithms have been effectively employed to forecast the impact of climate change on groundwater quality and variables. Key models include Neural Networks (such as ANN, GRU, LSTM, and RBFNN), renowned for their pattern recognition capabilities in predicting groundwater levels and quality [54,134,137,138]. Support Vector Machines (SVM) [54,134] and Random Forest (RF) [135,138] are widely used for their robust analysis in groundwater level forecasting and storage mapping. The Extreme Learning Machine (ELM), often enhanced with metaheuristic algorithms, has shown notable success in groundwater fluctuation studies [127]. Deep Learning approaches like 1-D CNN and Graph WaveNet are utilized for complex predictions under varying climate scenarios [139,140]. Additionally, the Adaptive Neuro-Fuzzy Inference System (ANFIS), particularly when integrated with algorithms like Ant Colony Optimization, has improved groundwater prediction accuracy [141,142]. Hybrid and Ensemble Models, combining multiple ML/AI techniques, have also proven effective in providing more accurate and reliable predictions in multifaceted groundwater analysis scenarios [140,143,144]. Fig. 3 presents the number of reviewed studies employing different ML/AI algorithms for predicting/forecasting/modelling different groundwater variables. In Fig. 3, the groundwater variables acted as the response variable for the respective ML/AI models used for various studies reviewed in this research. Therefore, the time step specificity in terms of these variables (for example daily, monthly, yearly) was dependent on the research aim, objectives and the data availability within the reviewed studies.

#### 3.2.1 Neural networks

Neural networks, with their layered structure comprising input, hidden, and output neurons, have become essential in analyzing groundwater level (GWL) fluctuations. Artificial neural networks (ANN), a prevalent ML method [[145], [146], [147]], have shown diverse effectiveness based on the chosen input variables. Enhancements in neural network methods, particularly the Extreme Learning Machine (ELM), have been achieved through integration with metaheuristic algorithms. Studies by the authors of Ref. [148,149] illustrate that ELM, when combined with approaches like the Jellyfish Search Optimizer, markedly improves GWL predictions. Notably, the inclusion of GWL itself as an input variable, alongside traditional climatic factors like precipitation and temperature, significantly bolsters prediction accuracy [55].

Other neural network variants, such as Bayesian artificial neural networks (BANN) and Gated recurrent unit (GRU) neural networks, have been employed using a range of climatic inputs to assess GWL. Authors of Ref. [132] compared ANN's performance with other models like Support Vector Regression (SVR), k-Nearest Neighbor (k-NN), and Random Forest (RF), finding ANN most effective in GWL simulation. Adaptive neuro-fuzzy inference systems (ANFIS) initially showed limited success in GWL prediction, but their efficacy improved considerably when combined with algorithms like ant colony optimization, as demonstrated by Ref. [141].

In comparing various neural network-based ML methods, the Radial Basis Function Neural Network (RBF) and hybrid artificial neural network (HANN) models emerged as particularly adept at forecasting GWL changes based on climatic variables. The HANN model, integrating machine learning with spectral analysis, proved highly effective in responding to irrigation demands. The authors of Ref. [150] utilized convolutional neural networks (1-D CNN) for evaluating GWL under different representative concentration pathway (RCP) scenarios, observing more pronounced decreases under RCP8.5. Conversely, authors of Ref. [151] used Graph WaveNet for GWL forecasting, outperforming models like LSTM and GRU.

Long Short-Term Memory (LSTM) networks have shown proficiency in real-time GWL forecasting, particularly in flood-prone areas, although their effectiveness varies geographically. Authors of Ref. [151] applied a Bidirectional Long Short-Term Memory (BLSTM) model for long-term GWL prediction, achieving optimal results with a blend of BLSTM and a single LSTM layer. Authors of Ref. [153] developed a deep belief network (DBN) for short-term GWL forecasting, influenced by local and global climate variables. The model's accuracy improved for longer lead times with a unique re-injection procedure. ANN techniques have also been extended to groundwater discharge forecasting and quality assessment. Authors of Ref. [131] employed ANN for total discharge prediction across the Korean Peninsula, integrating various data sources. In groundwater quality, an ensemble including ANFIS, SVM, and ANN was used, with ANN excelling in coastal aquifer vulnerability assessments. Table 1 provides the reviewed research that showcased outperforming Neural Network models for groundwater variables.

**Table 1:** Reviewed research with outperforming Neural Network models for groundwater variables.

Reference	Location	Method	Input Variable	Response variable	Recommended method/model
[154]	USA	Hybrid artificial neural network (HANN) mode	P, T, streamflow, irrigation demand, ENSO, Pacific Decadal Oscillation, North Atlantic Oscillation	GWL	hybrid artificial neural network (HANN) mode
[155]	USA	Long Short-term Memory (LSTM) networks and Recurrent Neural Networks (RNN)	GWL, P, and sea level data	GWL	LSTM
[156]	India	ANN, GP (Genetic programming), SVM and ELM(Extreme learning machine)	GWL, P	GWL	ELM
[131]	Korea	ANN	Terrestrial water storage change, P, SM, T	Discharge	ANN
[157]	USA	Gated recurrent unit (GRU) neural network	Streamflow, and catchment attributes (climate index, soil, and geological characteristics)	GWL	GRU
[152]	India	Bidirectional Long Short-Term Memory (BLSTM) model	P, T, ET, RH	GWL	BLSTM
[132]	Morocco	SVR, KNN, ANN, RF	P, river flow, ET	GWL	ANN
[99]	Australia	RF, ANN, LSTM	TWS anomalies, P, T, GWL	GWL	LSTM
[158]	Iran	ANFIS, SVM, and ANN	Hydraulic conductivity, GWL, distance from the shore, impact of the existing status of seawater intrusion in the region, and thickness of the aquifer	TDS	ANN
[153]	Africa	Deep Belief Network (DBN)	GWL, Terrestrial Water Storage, P, T, ENSO, North Atlantic oscillation, Atlantic multi-decadal oscillation, Indian ocean dipole, Pacific decadal oscillation	GWL	DBN
[141]	Iran	adaptive neuro-fuzzy inference systems (ANFIS) and metaheuristic algorithms, such as genetic algorithm (GA), particle swarm optimization (PSO), ant colony optimization for continuous domains (ACOR), and differential evolution (DE)	GWL, P, T, E, River flow	GWL	ANFIS-ACOR
[150]	Germany	1D-CNN	P, T	GWL	1D-CNN
[148]	Bangladesh	ELM and Optimization algorithms: Genetic algorithm (GA), Particle swarm optimization (PSO), Grey wolf optimization algorithm (GWO), Whale optimization algorithm (WHO), Harris hawks optimizer (HHO), Jellyfish search optimizer (JFO)	P, T	GWL	ELM-JFO
[151]	Columbia	Graph WaveNet (part of graph neural network)	P, T, VP, shortwave radiation, and day length	GWL	Graph WaveNet
[149]	Iran	GA-ANN and ICA-ANN hybrid models and ELM and ORELM models	stream flow, water depth and P	GWL	ORELM
[142]	Iran	MLP, ANFIS, Radial Basis Function Neural Network (RBFNN), SVM	GWL, P, T, ET	GWL	RBFNN
[159]	Barbados	Deep Neural Networks, Elastic-Net Regression, Generative Adversarial Neural Networks	GW pumping, recharge, P	GWL	–
[55]	Italy	LSTM, Non-linear Autoregressive Neural Network (NARX), CNN	P, T	GWL	LSTM

#### 3.2.2 Random forest and decision tree

Decision trees (DT) are versatile tools for classification and regression in predictive modeling, using a tree structure where nodes represent attribute choices and leaves indicate class labels or numerical values. Random Forests (RF), like DTs, combine multiple decision trees for robust predictions. The authors of Ref. [160] employed an ML-based RF regressor to comprehensively analyse global groundwater resources. Table 2 highlights studies where RF and DT models excelled, identifying influential hydrological and terrestrial drivers, predicting groundwater variations, and investigating land use impacts. The authors of Ref. [161] used ML-based regression to correlate future groundwater levels with temperature and precipitation, finding a strong correlation with these climatic variables.

**Table 2:** Reviewed research with outperforming Decision Tree and Random Forest models for groundwater variables.

Reference	Location	Method	Input Variable	Response variable	Recommended method/model
[168]	USA	BRT	P, LST, NDVI, SM, discharge anomaly, lithology, transmissivity, TWSA, GWLA	GWLA (groundwater level anomaly)	BRT
[169]	USA	BRT	TWSA, vegetation coverage, P, T, streamflow, Snow Water Equivalent, SM, and Plant Canopy Water, Drift Thickness and Aquifer Characteristics	GWLA	BRT
[177]	Iran	Cubist, RF, SVM, and Bayesian ANN	Elevation, slope, plan curvature, profile curvature, P, piezometric depth, distance from residential, distance from the river, Sodium, Potassium, and TWI	Nitrate	RF
[179]	Mali	SVC, logistic regression (LRG), DRT, RFC, KNN, LDA, NBA, MLP, Ada-boost classifier (ABC), quadratic discriminant analysis (QDA), gradient boosting classification (GBC), and Gaussian process classifier (GPC)	Lineament density, Lineament distance, Proximity to surface water (permanent or intermittent), Landforms, density, Topographic wetness index, NDVI, Soil, Land use/land cover, P	Groundwater potential zone	RF and SVM
[171]	Iraq	RF and KNN	elevation, slope, curvature, aspect, aquifer transmissivity, specific storage, soil, geology, transmissivity, specific storage, groundwater depth and groundwater quality	Groundwater storage and water quality	RF
[172]	Iraq	ANN, Decision Jungle (DJ), Averaged Perceptron (AP), Bayes Point Machine (BPM), Decision Forest (DF), Locally-Deep SVM (LD-SVM), BDT, Logistic Regression (LG), and SVM	aquifer saturated thickness, P, hydraulic conductivity, depth to groundwater, specific yield, elevation, density, soil, LULC, and distance to faults	Groundwater potential zone	DJ and BDT
[180]	Pakistan	RF, ANN	TWS, SM, surface runoff, canopy water, T, ET, P	Storage	RF
[132]	Morocco	Adaboost, RF, ANN, and SVR	EC, T, and pH	Ground water quality	Adaboost and RF
[164]	Denmark	ANN, SVM, RF	Clay content, Depth to clay occurrence, Soil drainage class, Soil type, DEM, Topographic wetness index, Slope, incoming solar radiation, horizontal distance to nearest waterbody, vertical distance to nearest water body, imperviousness, P, T	GWL	RF
[166]	India	multi-linear regression (MLR) and RF	P, ET, BE, SM, surface runoff, subsurface runoff, canopy water storage and canopy water evaporation	GWL	RF
[181]	Iran	Boosted generalized additive model (GamBoost), AdaBoost, Bagged classification and regression trees (Bagged CART), and RF	elevation, slope, aspect, curvature, TPI, TRI, valley depth, drainage density, distance from stream, P, TWI, soil order, lithology, distance from fault, and land use	Groundwater potential zone	RF
[165]	China	RF, Extra Tree Regressor (ETR), Adaptive Boosting Regressor (ABR), and Gradient Boosting Regressor (GBR)	TWSA, SM, ET, T, GWL	GWL	RF
[140]	Iran	boosted tree (BT), artificial neural network (ANN), deep learning neural network (DLNN), deep learning tree (DLT), and deep boosting (DB).	DEM, altitude, slope, aspect, plan curvature, profile curvature, drainage density, distance from the river, distance from the fault, land use, lithology, soil type, P, stream power index (SPI), and topographic wetness index (TWI)	GWL	DB
[160]	Global	DT, RF	Elevation, Slope, P, ET, T, LC	GWL	RF
[182]	China	RF, SVM, LR	AET, Drought index, PET, P, T, Elevation, Slope, Influence, NDVI	Arsenic	RF
[173]	Mali	SVM, linear support vector machines (LVC), logistic regression (LRG), the decision tree classifier (DTC), the random forest classifier (RFC), KNN, linear discriminant analysis (LDA), Gaussian naïve Bayes classification (NBA), multi-layer perceptron neural network (MLP), AdaBoost classifier (ABC), quadratic discriminant analysis (QDA), gradient boosting classification (GBC), the Gaussian process classifier (GPC), the ridge classifier (RID), stochastic gradient descent linear classifier (SGD), perceptron (PER), passive-aggressive classifier (PASSI), the nu-support vector classifier (nuSVC) and the extra trees classifier (ETC)	Clay content, Curvature, Saturated thickness Water table depth, Distance from channels, P, Drainage density, Elevation (DEM), NDVI, NDWI (normalized difference water index), Slope, SPI (stream power index), TWI	Groundwater flow	DTC and RFC
[183]	Denmark	RF	P, T, sea-level, PET	GWL	RF
[163]	Ireland	Random Forest regressor	Height above Nearest Drainage (HAND) DEM, depth to bedrock, peat, recharge coefficient, groundwater vulnerability, subsoil permeability	GWL	Random Forest regressor
[184]	Ghana	Hybrid model (HM) of Bayesian random forest (BRF), Bayesian support vector machine (BSVM), and Bayesian artificial neural network (BANN)	P, WS, RH, SH, T, sunlight, and SM	GWL	BRF
[185]	USA	RF	P, T, TWSA, SM, GWSA, Saturated hydraulic conductivity, Texture, Percent Slope	Storage	RF
[161]	Iran	–	P, T	GWL	–
[170]	Turkey	RF	TWSA, DEM, SM, snow water, ET, P, water runoff	Storage	RF
[137]	Iran	RF, SVR, MLP	P, ET, T, NDVI	Storage	RF
[167]	Australia	RF	P, PET, streamflow, forest cover, relief, slope and available soil water holding capacity in topsoil	Discharge	RF

The authors of Ref. [162] noted precipitation's highest correlation with future groundwater level fluctuations. The authors of Ref. [163] used RF to assess groundwater memory and vulnerability, emphasizing factors like surface topography and overburden thickness (>10 m) and the authors of Ref. [164] found RF outperformed ANN in groundwater level prediction. RF and RF-based ML methods also downscaled GRACE-derived water storage changes. Among various ML methods, RF consistently displayed the best accuracy [165]. The authors of Ref. [166] emphasized the impact of specific predictor variables, such as evapotranspiration, land surface temperature, soil moisture, and subsurface runoff.

RF proved effective for assessing groundwater drought at different timescales [167]. It revealed a lag of over 12 months between precipitation and groundwater drought response. Catchments with higher drought resilience exhibited lower resistance and vulnerability, influenced by climate and physical properties. ML methods have been used to downscale groundwater level (GWL) data. The authors of Ref. [168] employed a boosted regression tree (BRT) model, achieving spatial GWL anomaly predictions in the USA and the authors of Ref. [169] used a similar BRT model, highlighting the impact of GRACE total water storage anomaly (TWSA).

In Turkey, the authors of Ref. [170] used RF to explore local-scale groundwater storage fluctuations, consistently outperforming other ML methods. RF enhanced spatial representation and identified GWSA hotspots. Groundwater potential mapping, critical for assessing water availability, used ML methods. The authors of Ref. [171] found RF most accurate, with groundwater depth, elevation, transmissivity, specific storage, and soil as key factors. A similar study in Iraq by Ref. [172] favored DJ and BDT models, emphasizing aquifer thickness, rainfall, hydraulic conductivity, depth to groundwater, specific yield, and elevation.

In Mali, the authors of Ref. [173] found tree-based algorithms consistently outperformed SVM and ANN. Similar to this, Ref. [138] favored Gradient Boosted Decision Trees for groundwater potential mapping, suggesting ML potential to surpass traditional methods. Ref. [174] highlighted soil type and land use/land cover as crucial features for groundwater potential mapping. Ref. [175] used ML models, including Adaboost and RF, to predict groundwater quality for irrigation, finding superior performance compared to SVR and ANN. Other studies focused on groundwater arsenic and nitrate levels, with RF consistently outperforming other ML methods [176,177]. Ref. [178] used RF to predict salinity concentrations, identifying precipitation, recharge, evaporation, proximity to the ocean, and fractured rocks as significant factors.

#### 3.2.3 Support vector machine (regression)

The Support Vector Machine (SVM) differs from Artificial Neural Networks (ANNs) by relying on Structural Risk Minimization (SRM) instead of Empirical Risk Minimization (ERM) [186]. This divergence from ERM helps prevent the model from getting trapped in local minima and overfitting. SRM optimizes both empirical error and model complexity simultaneously, enhancing the SVM's capacity for effective generalization in various fields when handling classification or regression tasks. In recent times, SVMs have found application in forecasting future changes in water resources, as shown in Table 3. For example, Ref. [157] introduced a hybrid model (HM) consisting of Bayesian Random Forest (BRF), Bayesian Support Vector Machine (BSVM), and BANN to automate logical inference and decision-making in business intelligence related to groundwater management. Among the variables considered, precipitation, temperature, and sunlight were found to have the most significant impact on Groundwater Level (GWL) [157].

**Table 3:** Reviewed research with outperforming SVM and SVR models for groundwater variables.

Reference	Location	Method	Input Variable	Response variable	Recommended method/model
[37]	USA	SVM	GWL, P, T, and solar radiation	GWL	SVM
[181]	Iran	Flexible discriminant analysis (FDA), mixture discriminant analysis (MAD), BRT, MARS, RF, SVM	levation, slope, aspect, curvature, TPI, distance from stream (DFS), P, E, TWI, discharge, GWL, distance from fault (DFF), lithology, soil order, landuse, and distance from road (DFR)	salinity	SVM
[133]	Iran	Multivariate adaptive regression spline (MARS) and SVM model	elevation, aspect, slope, plan curvature, profile curva- ture, distance from fault, distance from road, distance from river, drainage density, land use, lithology, soil, SPI, TWI, annual pre- cipitation, precipitation of coldest month, precipitation of cold- est season, precipitation of wettest season	Groundwater potential zone	SVM
[141]	Iran	support vector regression (SVR), and least-squares SVR (LSSVR)	GWL, P, T	GWL	LSSVR

Following a similar concept, Ref. [37] reported that implementing the SVM algorithm along with data assimilation (DA) techniques, and considering factors such as precipitation, solar radiation, air temperature, and infrared surface temperature, could predict changes in GWL with a lead time of up to 3 months. A recent study conducted in Iran by the authors of Ref. [133] for assessing groundwater potential modeling used two ML models, Multivariate Adaptive Regression Spline (MARS) and SVM, along with two hyperparameter optimization algorithms, Random Search (RS) and Bayesian Optimization, to fine-tune the SVM model's parameters. In this study, an analysis of variable importance in the groundwater potential model revealed that elevation, precipitation in the coldest month, soil, and slope variables played a crucial and highly significant role.

In a comparatively earlier study by the authors of Ref. [181], various machine learning models, including Flexible Discriminant Analysis (FDA), Mixture Discriminant Analysis (MAD), Boosted Regression Trees (BRT), MARS, Random Forest (RF), and SVM, were used for groundwater salinity mapping. The results demonstrated that the SVM model outperformed the other models, and soil order, groundwater withdrawal, precipitation, land use, and elevation were identified as the most significant variables contributing to groundwater salinity mapping. Furthermore, when combined with MODFLOW [187], improved Support Vector Regression (SVR) models showed better accuracy than standard SVR models, highlighting precipitation as the primary climatic variable responsible for future GWL fluctuations [141].

#### 3.2.4 Hybrid and ensemble ML methods

Hybrid and ensemble ML methods represent powerful approaches that leverage multiple ML techniques and models to enhance prediction accuracy and robustness [188]. While some studies have employed single ML methods for groundwater level (GWL) assessment, recent research as per Ref. [43] ventured into the realm of employing multiple shallow ML models, including feed-forward neural networks (FFNN), support vector regression (SVR), adaptive neuro-fuzzy inference system (ANFIS), and even deep learning-based long short-term memory (LSTM) networks. They sought to forecast future GWLs and assess the impact of climate change on GWL. Similarly, a study by the authors Ref. [143] introduced a novel hybrid ensemble modelling framework encompassing locally weighted linear regression (LWLR) and four Gaussian process regressions (GPRs) to predict GWL in drought-prone regions. This approach highlighted the significant influence of various factors on accurate and dependable local-scale GWL estimation. Table 4 shows the various hybrid ML models for predicting/forecasting groundwater variables.

**Table 4:** Reviewed research with outperforming hybrid and ensemble models for groundwater variables.

Reference	Location	Method	Input Variable	Response variable	Recommended method/model
[181]	Iran	Stochastic Gradient Boosting (StoGB), Rotation Forest (RotFor), and Bayesian Generalized Linear Model (Bayesglm)	elevation, slope, aspect, curvature,TWI, distance from stream (DFS), distance from lake (DFL), distance from fault (DFF), depth of groundwater (DTGW), groundwater withdrawal (GWW), decline of groundwater level (DGWL), E, P, land use, lithology, and soil type	Salinity	RotFor and Bayesglm
[194]	Morocco	RF, LR, DT and ANNs	Elevation, Aspect, Slope, Curvature, Profile curvature, Plan curvature, Convergence, TWI, SPI, TRI, MeRugNu, MRRTF, MRVBF, LS, Lithology, Distance to Faults, Faults Density, Distance to lineaments, Lineaments density, Distance to rivers, Rivers density, NDVI LULC, rainfall	Groundwater potential zone	RF-LR-DT-ANN ensemble model
[138]	India	(ARZ ensemble) Automatic Multilayer Perceptron (AutoMLP), RF, and ZeroR	altitude, topographic wetness index, slope, slope aspect, curvature, P, lithology, land use, and proximity to rivers	Groundwater potential zone	–
[191]	USA	ANN, SVM, response surface regression	stream inflows, surface water diversions, P, ET, land use acreages	Storage	Ensemble model
[140]	Iran	boosted tree (BT), artificial neural network (ANN), deep learning neural network (DLNN), deep learning tree (DLT), and deep boosting (DB).	DEM, altitude, slope, aspect, plan curvature, profile curvature, drainage density, distance from the river, distance from the fault, land use, lithology, soil type, P, stream power index (SPI), and topographic wetness index (TWI)	GWL	DB
[143]	Bangladesh	locally weighted linear regression (LWLR) and four Gaussian process regressions (GPRs), e.g., polykernel, Pearson universal kernel (PUK), radian basis function (RBF), and normalized poly kernel.	P, T, SM, NDVI, Indian Ocean Dipole (IOD), Southern Oscillation Index (SOI), and population growth rate	GWL	LWLR-GPR-PUK
[189]	China	SVM, RF and multiple perceptions (MLP)	GWL, T, P, WS and sunshine hours	GWL	stacking ensemble model
[192]	Iran	Group method of data Handling (GMDH)	SM, ET, T, plant canopy Surface Water changes	GWA	–
[193]	Iran	frequency ratio (FR), RBF, index of entropy (IOE), evidential belief function (EBF) and fuzzy art map (FAM)	locations of calibration wells, Altitude, slope, slope aspect, curvature, TRI, TWI, lithology, soil, distance to fault, distance to stream, rainfall, drainage density, flow direction, land use	Groundwater potential zone	FR-FAM and FR-RBF ensemble models
[139]	Saudi Arabia	GLM, DBM, and DNN, XGBoost-based SHAP (SHapley Additive exPlanations)	Total dissolved solids (TDS), electrical conductivity (EC), turbidity (Turb), pH, iron (Fe), total hardness (TH), chloride (Cl), nitrate (NO3), sulfate (SO4), manganese (Mn), copper (Cu), zinc (Zn), chromium (Cr)	WQI	Stacking model (GLM, DBM, and DNN)
[195]	India	Novel ensemble approach ANN-MLP, RF, M5 prime (M5P) and sSMOReg	elevation, slope, aspect, drainage density, P, water table depth, lineament density, land use land cover, geomorphology, and soil types,	Groundwater potential zone	–
[190]	Canada	Generalized Additive Model for Location, Scale and Shape (GAMLSS)	Discharge, P, T, antecedent wetness	Discharge	–
[43]	Iran	FFNN, SVR, and ANFIS and their ensemble, as well as DL-based LSTM	GWL, P, T	GWL	Ensemble method

Ensemble ML methods have consistently demonstrated improved accuracy in predicting GWL, as shown in Ref. [189], who found that stacking ensemble models outperformed individual ML models. Their research emphasized rainfall as the most influential factor affecting GWL. In addition to ensemble methods, hybrid models such as the K-Nearest Neighbor-Random Forest (KNN-RF) have shed light on the key factors influencing GWL variations. The authors of Ref. [140] employed hybrid deep learning algorithms, including neural network decision trees and boosting methods, to assess groundwater potential maps using a range of input variables, from climatic data to topographic features. Their study identified altitude, rainfall, distance to fault lines, and soil types as primary factors in groundwater potential modelling, ultimately recommending the use of deep boosting (DB) models for superior performance.

Investigating the impact of climate change on monthly groundwater flow trends across Canada, authors of Ref. [190] turned to generalized additive models for location, scale, and shape (GAMLSS), a regression-type model that incorporates parametric distribution for response variables. Their analysis of streamflow data spanning three decades highlighted the influence of climate predictors such as precipitation, temperature, and antecedent wetness on baseflow trends. It revealed antecedent wetness as a frequently occurring climate predictor during trend analysis and pointed to warmer temperatures and increased snow cover as contributing factors to augmented baseflow, potentially linked to changes in snowmelt patterns. Interestingly, some baseflow trends were not associated with climate predictors, suggesting human activities may play a role in altering baseflow patterns. Few studies have explored ML approaches for assessing the impact of climate change on groundwater storage. In USA, a Bayesian ML ensemble method that included artificial neural networks (ANN), support vector machines (SVM), and response surface regression was employed to quantify model uncertainty in predicting changes in groundwater storage. The research mentioned in Ref. [191] highlighted that, contrary to climatic parameters, groundwater pumping for agricultural irrigation predominantly influenced changes in groundwater storage across the region.

Furthermore, the authors of Ref. [192] conducted a study on forecasting groundwater anomaly (GWA) in terms of storage, utilizing group method of data handling (GMDH) and three input variables: soil moisture, average temperature, and evapotranspiration. Their findings indicated that these variables provided the most favourable performance compared to other models.

Additionally, the study suggested that consequent changes in terrestrial water storage anomaly could significantly reduce groundwater availability in the future. Iran has also seen several studies employing various ML methods for groundwater potential modelling, mapping, and prediction [133,181,193]. Ref. [193] focused on optimizing ML hybrid and statistical models for groundwater potential mapping, with models like radial basis function (RBF) and fuzzy art map (FAM) demonstrating high accuracy. However, limitations arose due to insufficient geological data and the absence of key groundwater parameters.

However, recently most studies have reported that the ensemble and hybrid ML models consistently outperformed individual models in predicting/forecasting groundwater variables incorporating the climate events. Their ability to integrate multiple ML techniques and account for complex interactions among influencing factors has advanced the accuracy and understanding of GWL, groundwater storage, and potential modelling. Nonetheless, ongoing research and data collection efforts are crucial to further refine these models and enhance their applicability in groundwater management and climate change impact assessment.

## 4 Climate change and groundwater condition in Ireland

**Fig. 4:**
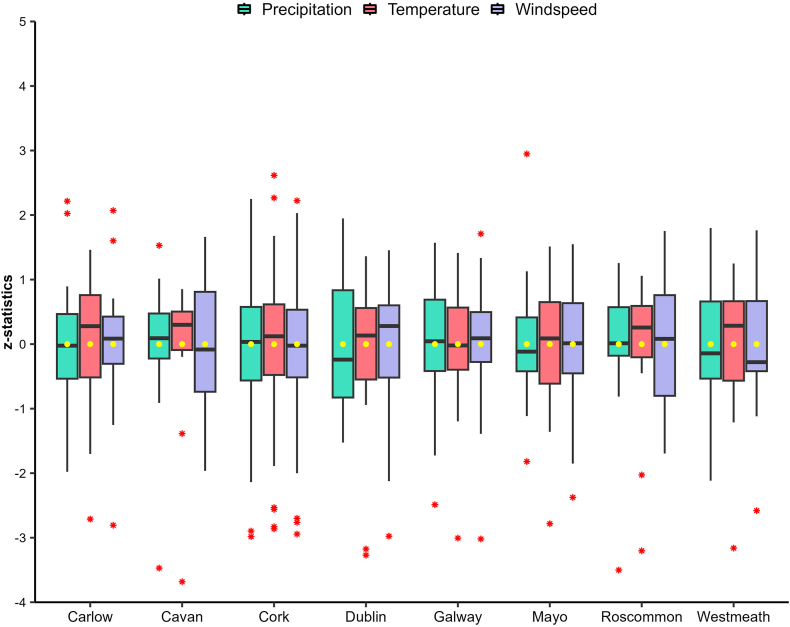
Comparison of precipitation, temperature and windspeed from year 2004–2022 for the selected counties.

Ireland is experiencing undeniable impacts of climate change, with a substantial increase in temperatures observed over the years. Between 1890 and 2008, Ireland witnessed a remarkable temperature rise of 0.7 °C, with a particularly pronounced surge of 0.4 °C occurring between 1980 and 2008 [[196], [197], [198]]. These escalating temperatures have instigated profound alterations in Ireland's natural environment, resulting in significant consequences for various aspects of the nation. Fig. 4 presents a comprehensive summary of statistical data pertaining to various climate variables across Ireland for the period spanning from 2004 to 2022.

One noteworthy outcome of these temperature shifts is the transformation of farming practices. The evolving climate has brought about shifts in growing seasons, fundamentally impacting the timing and nature of agricultural activities [199]. Furthermore, the higher temperatures have created conditions conducive to the presence of animals adapted to warmer climates, thereby exerting a profound influence on Ireland's agricultural landscape [200]. In addition to these temperature-related changes, Ireland has experienced a marked increase in the frequency and severity of storms in recent decades [201]. These extreme weather events pose multifaceted challenges to various sectors, including infrastructure, coastal regions, and emergency preparedness.

To underscore the evolving climate in Ireland, it is imperative to consider key climatic variables, notably precipitation, temperature, and windspeed. The Irish National Meteorological Service, known as Met Éireann, is responsible for monitoring and collecting various climatic variables' data. However, due to data inconsistency over the years, 8 counties were chosen wherein data for at least three climatic variables including temperature, precipitation and windspeed are available. Comprehensive data from Met Éireann, collected from 8 strategically selected stations across the country, provide concrete evidence of these climatic shifts (Fig. 4). These variables have been meticulously chosen for representation due to their pivotal role in comprehending the dynamic climate. The data analysis has been conducted for 8 carefully selected counties in Ireland, ensuring a comprehensive evaluation of these crucial climatic indicators across diverse regions of the country. This comprehensive assessment aims to shed light on the multifaceted impact of climate change on Ireland's environment and its implications for various sectors.

**Fig. 5:**
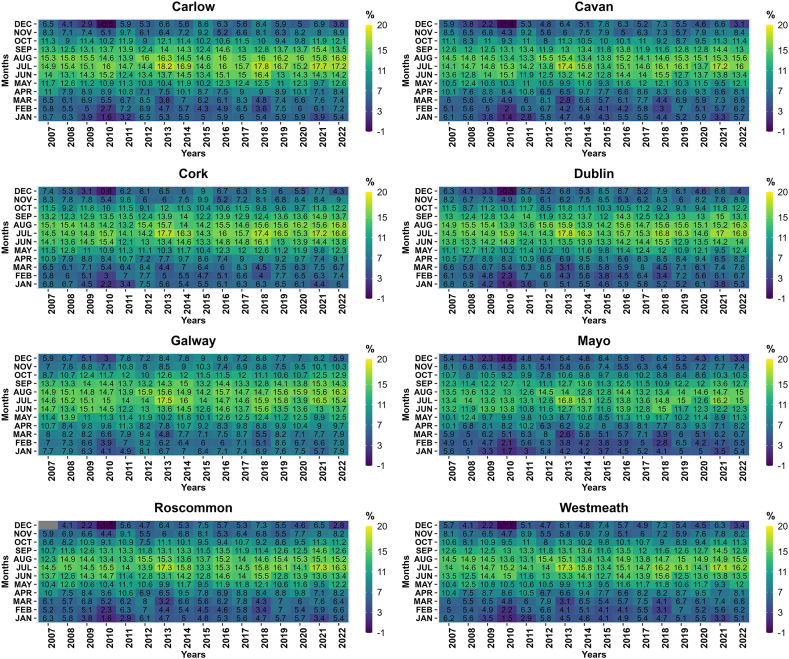
Temperature trend (yearly average) in the selected counties from 2007 to 2022.

As discussed earlier, these climatic variables serve as critical input parameters for evaluating their influence on groundwater dynamics. A prior investigation conducted in Ireland by the authors of Ref. [202], focusing on nine distinct catchments, aimed to assess the potential ramifications of climate change on hydrological processes. This study unveiled those alterations in mean winter and summer flows, as well as the annual maximum daily mean flow, exhibited variations contingent upon the unique characteristics of each catchment and the timing and magnitude of anticipated shifts in precipitation within these specific areas. Consistent findings were corroborated by a recent research endeavor conducted by the authors of Ref. [203]. Their analysis, which delved into long-term trends concerning daily extreme air temperature indices in Ireland, represented the inaugural evaluation of the frequency, duration, intensity, and geographic distribution of these indices. The outcomes underscored substantial increasing trends in both seasonal and annual maximum and minimum air temperatures across Ireland. Notably, the spring and autumn seasons demonstrated more pronounced temperature increases, aligning with global patterns elucidated in the Sixth Assessment Report of the Intergovernmental Panel on Climate Change (IPCC). This further underscores the substantial impact of climate change on temperature extremes. Another antecedent study, which focused on the development of future temperature scenarios in Northern Ireland, addressed specific climate-related issues. The research indicated that the future downscaled scenarios depicted a significant warming trend across all sites and seasons [204]. Furthermore, the influence of climate change scenarios extends to maximum lake surface temperatures, as evidenced by a comprehensive evaluation of 50 years of observational data [205]. Fig. 5 elucidates the temporal trends in temperature across various counties in Ireland over a 16-year period.

While various studies in Ireland have assessed temperature changes, research efforts have also been dedicated to evaluating the repercussions of climate change on precipitation within the mid-century period spanning from 2041 to 2060. This research has indicated significant decreases in mean annual, spring, and summer precipitation amounts by the mid-century timeframe [206]. Furthermore, the study has underscored a noteworthy increase in the frequency of heavy precipitation events, accounting for approximately 20 %, during the winter and autumn months. It is worth noting that such alterations in climate have profound implications for biodiversity. Primary evidence of climate change's influence on biodiversity in Ireland primarily stems from a limited set of phenological studies. These studies reveal patterns of earlier leafing, bird migration, and insect appearance, which appear to be, at least partially, linked to the rising spring temperatures [207].

**Fig. 6:**
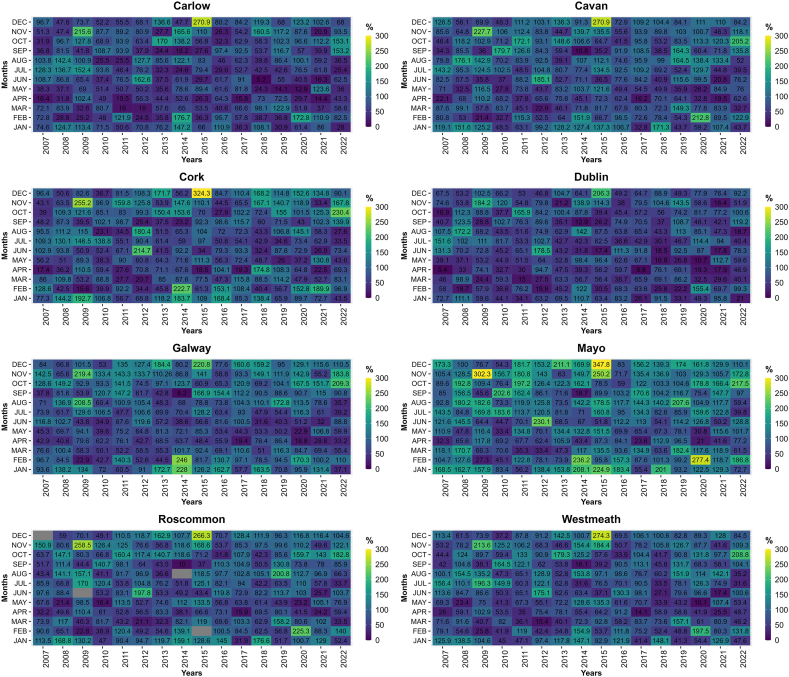
Precipitation (yearly average) patterns in the selected counties, Ireland from 2007 to 2022.

Another significant impact of climate change pertains to extreme hydro-climatic events within specific regions. For instance, in research focusing on the potential consequences of climate change on a river catchment within an urban area, the results indicated a potential increase of up to 12 % and 16 % in flood quantiles for 50-year and 100-year return periods, respectively (Basu et al., 2022). Similar findings were reported by the authors of Ref. [208], where modeling techniques were employed to examine the effects of sea-level rise on coastal inundation depth. This assessment considered both the present period and the year 2100, contemplating two Representative Concentration Pathways (RCP 4.5 and RCP 8.5). The results underscored substantial challenges ahead, with an estimated increase of approximately 26 % and 67 % in the number of administrative units categorized as being at very high risk by the end of the century under RCP 4.5 and RCP 8.5, respectively. Fig. 6 presents a visual representation of the precipitation trends across various counties in Ireland over a 16-year period, further highlighting the changing climate dynamics in the region.

In addition to temperature and precipitation, windspeed has also been significantly influenced by various climate change scenarios, including the North Atlantic Oscillation [209,210]. According to the Irish Ocean Climate and Ecosystem Status Report 2023, during extreme climatic conditions where both the North Atlantic Oscillation (NAO) and the East Atlantic (EA) patterns simultaneously exhibit either positive or negative phases, wind speed anomalies over Ireland are diminished compared to situations where only the NAO is in a positive or negative phase. Conversely, when the NAO and EA patterns have opposite signs, wind speed anomalies are amplified in the mid-Atlantic and Ireland [211].

In a study conducted by the authors of Ref. [212], the inshore wave climate at 63 locations across the United Kingdom and Ireland from 1980 to 2017 was analyzed. The investigation revealed that 73 % of these locations exhibit a directionally bimodal wave pattern. Furthermore, the study explored the relationships between six prominent atmospheric indices and both the total and directional winter wave power (peak spectral wave direction) at all the studied sites. Notably, this research marked the first time that the leading winter-averaged atmospheric indices were shown to hold significant explanatory power for directional wave climates.

**Fig. 7:**
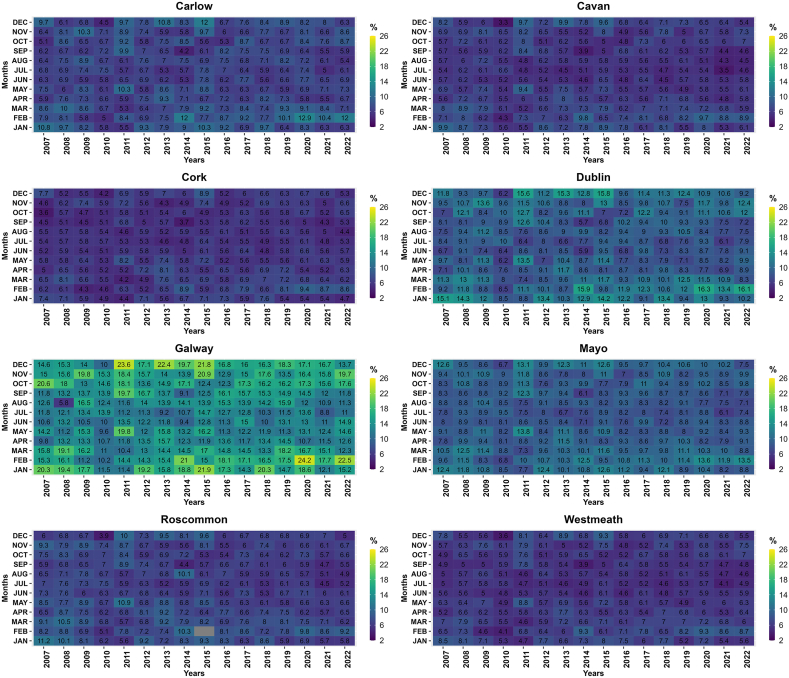
Windspeed (yearly average) patterns in the selected counties, Ireland from 2007 to 2022.

Addressing the impact of climate change on wind energy in Ireland, Ref. [213] concluded that these impacts display a clear seasonality. There is a more substantial decrease during summer, projecting a reduction of less than 6 %. In contrast, winter was expected to experience a slight increase in wind energy (up to 1.1 %). This seasonal variation led to higher intra-annual variability, potentially resulting in greater irregularity in wind energy production throughout the year. Fig. 7 visually represents the trends in windspeed across various counties in Ireland over a 16-year period, further emphasizing the evolving climate dynamics in the region.

### 4.1 Groundwater condition in Ireland

**Fig. 8:**
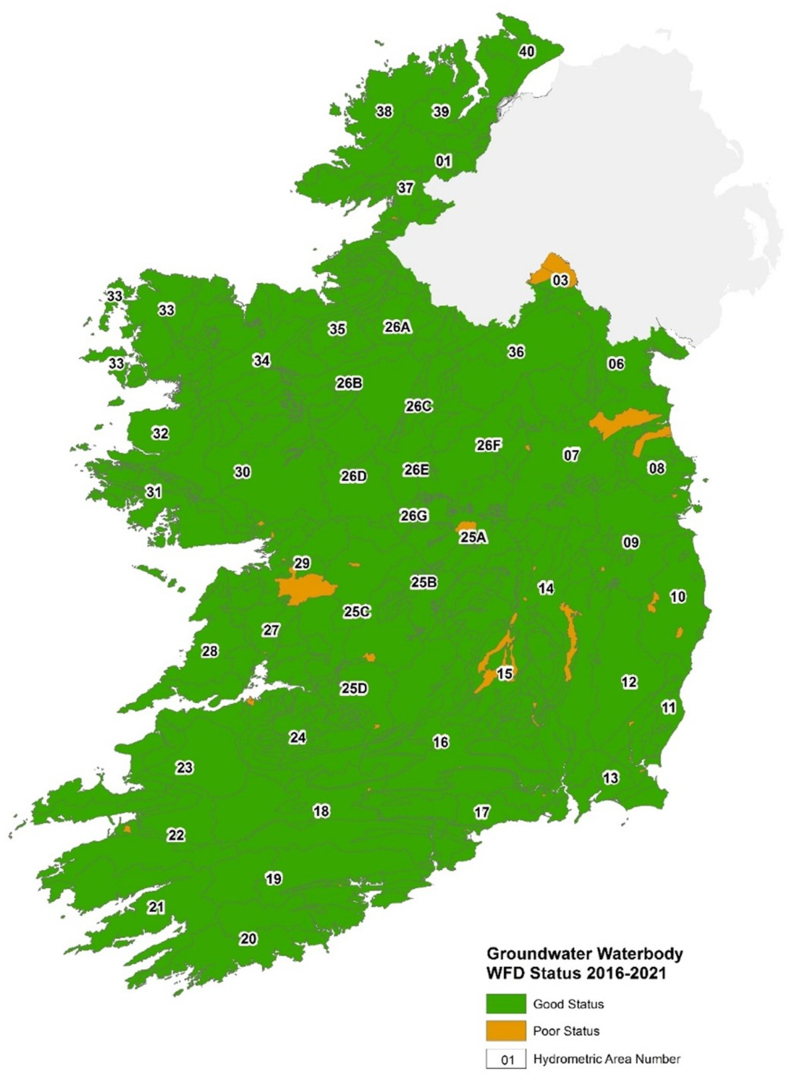
Groundwater waterbody in Ireland according to WFD status (2016–2022) Adopted from Ref. [218].

Groundwater plays a vital role as a water supply source in Ireland, accounting for approximately 25 % of water supplies [214]. This reliance on groundwater increases in rural areas where private wells serve households without access to public mains supplies [215]. Beyond direct water supply, groundwater also sustains river base flow and provides water to vital ecosystems such as fens and turloughs [216]. According to the Groundwater Waterbody Water Framework Directive (WFD) Status 2016–2021, nearly 92 % of groundwater bodies in Ireland maintain good chemical status, with most being in good quantitative status, indicating an adequate volume of water present in the groundwater body [217] (Fig. 8).

However, recent studies focusing on various groundwater variables have revealed that several counties in Ireland face groundwater-related risks in terms of both level and quality [163,219,220]. Additionally, anticipated changes in recharge patterns and increased exploitation of coastal aquifers in the future could potentially disrupt the delicate balance between recharge rates, shifting rainfall patterns, and contaminant accumulation in Ireland's groundwater reservoirs [163].

One study conducted in 2016 as mentioned in Ref. [221] investigated groundwater-level hydrographs from two Irish hillslope sites with hard rock aquifers. They applied correlation methods to understand how groundwater levels responded to rainfall and seasonal variations. The results suggested that direct groundwater recharge to the shallow and deep bedrocks on the hillslope was limited, and water-level fluctuations within these geological units were likely influenced by slow-flow rock matrix storage. In contrast, monitoring wells at the subsoil and soil/bedrock interface, as well as those in the shallow or deep bedrocks at the base of the hillslope, exhibited rapid responses to rainfall with little seasonal variations, implying direct recharge within these units. Additionally, statistical analysis revealed that low rainfall intensity (≤1 mm/h) had a more significant impact on groundwater recharge rates compared to higher rainfall intensity groups (>1 mm/h).

**Fig. 9:**
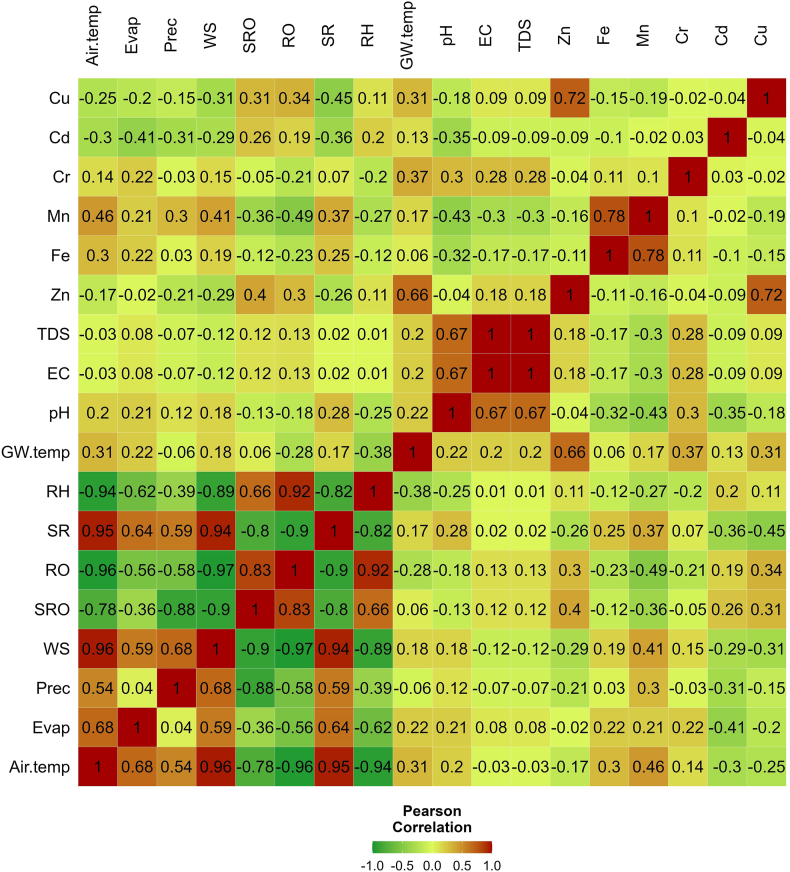
Correlation between climatic variables and groundwater quality variables in Cork (2022).

A preliminary correlation analysis of groundwater quality variables and climatic factors in County Cork is shown in Fig. 9. The groundwater quality data is taken from the Irish Environmental Protection Agency (EPA) monitoring database (https://www.catchments.ie/data), and the climate factor data are taken from https://cds.climate.copernicus.eu/cdsapp#!/dataset/reanalysis-era5-single-levels. The groundwater quality data is taken for 15 stations in Cork for the year 2022 (yearly average). The groundwater quality taken into consideration includes temperature, pH, electrical conductivity, TDS, and trace metals (zinc (Zn), copper (Cu), iron (Fe), manganese (Mn), chromium (Cr), cadmium (Cd)).Similarly, the yearly average climatic data is taken for 15 points in Cork and climate factors taken into consideration are air temperature, evaporation (evap), precipitation (prec), wind speed (ws), surface run-off (SRO), total run-off (RO), and relative humidity (RH). In our comprehensive analysis of environmental data, the identified correlation patterns offer valuable insights into the intricate interdependencies among crucial environmental factors, thereby enhancing our comprehension of climate change impacts on groundwater quality. The strong positive correlation between air temperature and solar radiation (0.948) underscores the amplifying effect of rising temperatures on solar energy absorption, potentially exacerbating evaporation rates and drought conditions, critical aspects of climate change impact assessment. Additionally, the negative correlation between air temperature and total run-off (RO) (−0.956) highlights the potential consequences of elevated temperatures, contributing to decreased freshwater availability—a critical concern in regions vulnerable to climate-induced water scarcity. Furthermore, the positive associations observed between heavy metal concentrations (e.g., Zn-Cu and Fe-Mn) may signify shared sources or transport mechanisms influenced by changing climate dynamics. These correlation findings provide invaluable insights for future research, guiding informed decision-making in climate change adaptation and groundwater resource management, essential aspects of addressing the multifaceted challenges posed by climate change. This further provides a base for carrying out further investigation on the impact of different climatic factors on groundwater quality variables using advanced statistical tools and ML/AI models.

Groundwater flooding risk has also been a subject of study as mentioned in Ref. [222] for mapping groundwater flooding risk. They observed various mechanisms, including backwater flooding of sinks, overland flow from overtopping sink depressions, high water levels in turlough basins, and surface ponding in local epikarst watersheds. Over the past few decades, there has been an increase in groundwater flood frequency, primarily in response to higher winter rainfall in the western part of Ireland. If this trend continues, extreme flooding events, like the one experienced in 2009, could become more frequent and permanent, necessitating re-evaluation of mitigation measures.

Furthermore, Ref. [163] explored the use of RF to simulate groundwater memory on a national scale, particularly focusing on drought susceptibility indicators. This study gains significance in light of the projected increase in the frequency and severity of droughts due to climate change. RF-based mapping of groundwater memory offers valuable insights into regions more or less vulnerable to drought and related stress. Areas with high memory are expected to have abstraction regimes less affected by meteorological droughts, offering greater reliability during dry periods. Conversely, regions with low memory may require enhanced measures to enhance resilience to drought.

Another study by the authors of Ref. [224] examined long-term trends in a lowland karst aquifer's dynamic, including spring discharge, groundwater levels, and groundwater flooding. The research found that these dynamics were influenced by precipitation patterns in the weeks and months leading up to peak water levels, which typically occur in late winter or early spring. This study projected significant increases in flood levels and longer flood durations, particularly at higher and more extreme flood levels. Consequently, what is currently considered high or extreme flooding may become more frequent in the future.

In terms of groundwater quality, a comprehensive evaluation conducted by the authors of Ref. [223] analyzed the distribution, spatial arrangement, and environmental implications of heavy metal content in stream sediments across 17 counties in Ireland. The study found that average concentrations of various trace metals and major/minor oxides were higher than background levels but below established sediment quality guidelines. Some regions, like Wicklow, Dublin, Mayo, Galway, and Cavan counties, exhibited hotspots with higher concentrations of these elements, linked to catchment geochemistry. Sites in Mayo and Donegal showed high contamination levels, influenced by factors such as lithology, topography, and human activities.

Additionally, nitrate pollution has been identified as a problem in Ireland's groundwater reservoirs. Ref. [219] compared the groundwater nitrate issue and mitigation strategies in highly impacted European Union countries such as Germany, Denmark, and Ireland. The study revealed an increasing trend, with more monitoring stations showing high nitrate concentrations (>25 mg/l) compared to previous reports. Nearly half (47 %) of all groundwater stations exhibited increasing nitrate concentrations from 2013 to 2020, with the southeastern part of the country particularly susceptible to nitrate leaching due to sandy soils. Agriculture, particularly the use of organic fertilizers, livestock excreta, and chemical fertilizers, was identified as the main source of nitrogen inputs. A recent study by the authors of Ref. [224] has also provided a comprehensive framework for assessing the impacts of hydro-climatic variables on surface water quality using ML/AI. This study can be used as a foundation by researchers for assessing the interrelationship between groundwater quality and changes in climatic factors.

Authors of Ref. [225] assessed the impact of the Summer 2018 drought on the quality of private groundwater in the southwest of Ireland, indicating hydrodynamic alterations. These findings emphasize the intricate interplay between climate change and groundwater conditions in Ireland, which warrants further research to understand correlations and plan future mitigation technologies. Depending on the specific groundwater variable and associated dataset, ML/AI models can be efficiently employed to investigate their applicability and replicability across different regions in Ireland, contributing to more informed groundwater management strategies.

## 5 Discussion

This study enhances the understanding of the interplay between climate change and different groundwater variables through a comprehensive overview and by drawing insights from the reviewed studies on the application of ML/AI models to assess the impact of climate change on different groundwater variables. It is one of the first studies that discusses the impact of different climatic variables on multiple groundwater variables, providing the readers with detailed information on the global research conducted in this domain.

In the reviewed studies on different groundwater variables, ANN ML methods emerged as the preferred choice among researchers due to their exceptional ability to recognize complex data patterns and facilitate feature learning. Neural networks consistently outperformed other ML methods, demonstrating lower Root Mean Square Error (RMSE) as compared to other models in the respective studies and superior prediction accuracy across various evaluation metrics [139,151,154,184]. Different models have been used in these studies, however, comparing the RMSE, ANN models exhibited least error in terms of RMSE. Readers can refer to the above-mentioned studies for specific details of the error percentage of different ML models as compared to ANN models. Furthermore, some studies explored the synergy of metaheuristic algorithms with ANNs to enhance predictive performance of different groundwater variables impact by climatic variables. The integration of algorithms like ELM and ANFIS with ANNs led to improved prediction results [148,156,158]. Precipitation has been consistently identified as a critical factor influencing GWL in many studies [142,148,149,151]. Neural network models proved their utility not only in GWL predictions but also in modeling other groundwater variables, including groundwater discharge [131] and groundwater quality [158].

Approximately 40 percent of the reviewed studies favored RF and DT based ML models for assessing changes in groundwater variables in response to various climatic factors. These models demonstrated effectiveness in enhancing the precision of spatio-temporal groundwater storage analysis using GRACE data [168,169]. RF models were also employed for mapping groundwater potential zones, taking into account variables such as groundwater depth, elevation, and transmissivity [171,172,179,181,194]. Additionally, RF and DT based models successfully assessed groundwater quality concerning contaminants such as arsenic [182] and nitrate [177] and played a pivotal role in predicting salinity concentrations, considering factors such as precipitation, groundwater recharge, proximity to the ocean, and geological features [178,226].

In addition to neural networks, RF, and DT based models, SVM based models were also employed widely in assessing the impact of climate change on ground water level, groundwater potential zone mapping, and groundwater quality. The SVMs transitioned from empirical risk minimization (ERM) to structural risk minimization (SRM) and found applications in predicting changes in water resources, particularly in groundwater management. SVM-based models identified precipitation, temperature, and sunlight as influential factors affecting GWL [37,141]. In groundwater potential zone modelling, SVM, in conjunction with MARS, utilized alongside hyperparameter optimization algorithms like random search (RS) and Bayesian optimization (BO) to fine-tune model parameters [133,226]. Key variables impacting groundwater potential included elevation, precipitation, soil, and slope [133]. SVMs exhibited superior performance in mapping groundwater salinity, with soil characteristics, groundwater withdrawal, precipitation, land use, and elevation being recognized as significant contributors [226].

While the individual models outperformed for predicting groundwater change due to climatic variables, hybrid and ensemble ML methods also gained prominence, offering improved prediction accuracy and robustness compared to individual ML models. Researchers harnessed the power of combining multiple shallow ML models, deep learning approaches, and ensemble techniques to achieve higher accuracy in GWL predictions and assess the effects of climate change [43,143,165,189]. These hybrid and ensemble methods exhibited potential for enhanced accuracy in GWL estimation, both on local and broader scales. The ensemble methods in the reviewed studies were particularly effective in groundwater potential mapping [[193], [194], [195],^227^], storage [191], discharge [190], groundwater quality assessment [139,226], shedding light on the key factors influencing these vital groundwater variables.

To extend the scope for future research, this study also underscores the undeniable impacts of climate change in Ireland, with significant temperature increase observed between 1890 and 2008, particularly during the period from 1980 to 2008. These temperature changes have had far-reaching effects on the natural environment, agriculture, and the frequency and severity of storms. Additionally, changes in precipitation, temperature, and windspeed data across various stations in Ireland have been pivotal in assessing their impact on groundwater. These climatic variables have played a crucial role in understanding the evolving groundwater scenario in Ireland, impacting both quantity and quality. Therefore, this research emphasizes the importance of further research to comprehensively understand the interplay between climate change and groundwater conditions in Ireland. As this review showcases that ML/AI techniques demonstrate their potential for mapping and predicting groundwater variables’ conditions based on climate change scenarios, researchers can choose appropriate ML/AI models based on their specific objectives and data availability, allowing for comparisons to identify the most accurate model for predicting changes in groundwater variables due to alterations in climatic factors. Data quality and reliability, along with uncertainty measures in ML/AI models, must also be considered. The availability and quality of data may vary across regions and variables, necessitating research on data interpolation, extrapolation, and imputation techniques to address potential challenges [228]. Ultimately, a thorough understanding of the relationship between climate change and groundwater conditions is essential for planning effective mitigation strategies, emphasizing the need for ongoing data monitoring and research in this field. This will also lead to efficient water resource management and effective science–policy interaction [229].

While ML/AI models offer promising tools for studying groundwater variables, it is crucial to acknowledge their limitations. These limitations include data quality and availability, data imbalance, overfitting and underfitting, model complexity and interpretability, limited insights into causality, data stationarity, transferability, data scaling and preprocessing, model selection and hyperparameter tuning, data uncertainty, and computational resource requirements. Researchers and practitioners should consider these limitations when implementing ML/AI approaches in groundwater studies and adopt appropriate strategies to address these issues. Collaborative efforts between hydrogeologists, climatologists, and data scientists can help overcome these challenges and lead to more accurate predictions of groundwater behaviour in response to changing climatic conditions.

## 6 Conclusions

Climate change exerts significant impacts on subsurface hydrology and geology, particularly influencing groundwater dynamics. The complex interplay between natural climate variability and human-induced disturbances results in variations in both the quantity and quality of groundwater across different spatial and temporal scales. Changes in precipitation patterns, evapotranspiration, glacier melting, and surface runoff contribute to alterations in the subsurface hydrological cycle. Understanding the intricate relationship between groundwater variables and climatic factors is a challenging endeavor, given the dispersed and time-delayed effects resulting from the wide range of groundwater residence times.

ML/AI methods have emerged as powerful tools for assessing and modeling changes in groundwater variables in response to climate change. This comprehensive review has examined various ML/AI techniques employed in recent studies, highlighting their effectiveness in modeling diverse groundwater parameters such as GWL, groundwater storage, discharge, quality, soil moisture, and the mapping of potential groundwater zones. Depending on the research objectives, different ML/AI methods have proven to be accurate and have shown potential for replication and scaling to different scenarios.

Notably, this review has focused on recent studies conducted since 2017, shedding light on the advancements in ML/AI-based groundwater assessment. The majority of the reviewed studies have concentrated on assessing the impact of climate change on GWL, including impact assessment, GWL forecasting, mapping potential groundwater zones based on GWL data, and downscaling GWL information. A recurrent finding in the literature is that hybrid and ensemble ML methods consistently outperform single ML/AI approaches in terms of accuracy and precision. However, the choice of input parameters significantly influences the accuracy of ML/AI models, as different studies use varying sets of climatic, topographic, land-use, and anthropogenic factors as inputs. Researchers have elucidated the individual contributions of these factors to changes in groundwater variables, assisting future researchers in selecting the most suitable ML/AI methods based on data availability.

Despite the promise of ML/AI models, one of the major limitations highlighted across studies is the unavailability of input data. Researchers emphasized the importance of data continuity for each ML/AI method, suggesting that readers consider the frequency of available data when selecting methods that require minimal input variables for optimal output accuracy.

Furthermore, the review contextualizes the groundwater conditions and projections in Ireland, emphasizing the potential impact of climate change. While some studies have assessed climate change effect on groundwater variables in Ireland, the use of ML/AI methods in this context remains relatively limited. Given the observed trends of deteriorating groundwater conditions in Ireland, it is anticipated that the impact of climate change on groundwater variables will become more significant in the future. However, it is essential to recognize that not all groundwater degradation can be attributed solely to climatic factors, as anthropogenic activities also play a substantial role.

In conclusion, this review provides a valuable foundation for future research endeavors. Researchers can leverage the insights gained from both ML/AI methods and available data for Ireland region to conduct studies projecting specific groundwater variables' responses to climate change factors. Data availability remains a critical factor in evaluating the effects of climate change on groundwater, and efforts should be directed towards improving data quality and monitoring practices. Additionally, prioritizing research into data interpolation, extrapolation, and imputation techniques will enhance data analysis using ML/AI methods, thereby contributing to a more comprehensive understanding of the relationship between climate change and groundwater conditions. Ultimately, this knowledge is crucial for effective groundwater management and climate change mitigation strategies.

## Data availability statement

Data will be made available on request.

## CRediT authorship contribution statement

**Apoorva Bamal:** Writing – review & editing, Writing – original draft, Visualization, Validation, Resources, Methodology, Formal analysis, Data curation, Conceptualization. **Md Galal Uddin:** Writing – review & editing, Writing – original draft, Validation, Supervision, Conceptualization. **Agnieszka I. Olbert:** Writing – review & editing, Supervision, Conceptualization.

## Declaration of competing interest

The authors declare that they have no known competing financial interests or personal relationships that could have appeared to influence the work reported in this paper.
