# Supplementary material for: Harnessing machine learning for assessing climate change influences on groundwater resources: A comprehensive review
Source: Heliyon. 2024 Aug 28;10(17):e37073. doi: 10.1016/j.heliyon.2024.e37073 (PMC11402946; doi:10.1016/j.heliyon.2024.e37073)
Supplement: Multimedia component 1 [file mmc1.docx]

**Supplementary materials:**

**Harnessing Machine Learning for Assessing Climate Change Influences on Groundwater Resources: A Comprehensive Review**

Apoorva Bamal^1,2,3,4,*^, Md Galal Uddin^1,2,3,4^, Agnieszka I. Olbert^1,2,3,4^

^1^School of Engineering, University of Galway, Ireland

^2^Ryan Institute, University of Galway, Ireland

^3^MaREI Research Centre, University of Galway, Ireland

^4^Eco-HydroInformatics Research Group (EHIRG), Civil Engineering, University of Galway, Ireland

*Corresponding author: Apoorva Bamal, PhD candidate, Civil Engineering, College of Science and Engineering, University of Galway, Ireland. Email: [a.bama1@universityofgalway.ie](mailto:a.bama1@universityofgalway.ie)

**Table S1.** Methods to assess impact of climate change on groundwater.

| S.No. | Methods | Description | Data | Advantages | Challenges | References |
| --- | --- | --- | --- | --- | --- | --- |
|  | Age dating | - Determine the timeframe of recharge, often by measuring the duration since recharge to: - Evaluate the susceptibility of aquifers. - Examine the mechanisms of saline intrusion. - Assess the suitability of a potential location for deep geological disposal | - Radioactive isotopes, such as ^14^C, with known and stable source concentrations. - Radioactive isotopes, such as ^3^H/^3^He, with varying source concentrations, and a daughter isotope that can be reliably associated with the parent species. - Conservative chemical species, such as ^85^Kr, SF_6_ that experience minimal decay and possess source concentrations. - H and O stable isotopes - Noble gas concentrations (Ne, Ar, Kr, and Xe). | - Provision of unique information compared to conventional hydraulic data, reducing ambiguity and uncertainty related to simulation parameters and stochastic parameter distributions and understanding system functioning. - Tracer data provide independently constrains numerical models, aiding in evaluating the conceptual basis of simulations | - Tracers provide average residence times and recharge rates, not specific age and date of recharge. - Averaging recharge rates ensures representative estimates but makes comparisons with other techniques challenging. - Tracer sampling and interpretation, as well as macroscopic dispersion and mixing, limit the precision of estimating groundwater residence times and recharge rates. | - Bethke & Johnson, 2008 - Newman et al., 2010 - Cartwright et al., 2017 - J. Zhang et al., 2021 |
|  | Chemical proxies | - Chemical proxies, such as stable isotopes of water and noble gases, are used to track changes in groundwater flow and recharge conditions related to climate change and surface environmental changes, as: - The chloride content of groundwater is used to infer changes in recharge conditions. - Stable isotopes of hydrogen and oxygen in water molecules are sensitive to evaporation and condensation |  | - Isotopic and noble gas studies provide insights into accurate changes in atmospheric circulation patterns, source area of precipitation, intensity, amount, and air temperature over longer time scales. - Meteorological stations measuring isotopes in precipitation offer direct information on shorter-term changes in weather and climate variables. - Elevated excess air content in groundwater serves as a proxy for significant water table fluctuations and high recharge variability |  | - Ashfaque, 2006 - Klump et al., 2008 - Ito & Forester, 2009 - Hall et al., 2012 - Gerber et al., 2017 |
|  | Electromagnetic methods | - Exploit differences in electrical resistivity and dielectric constant of subsurface materials; water has low resistivity and high dielectric constant compared to mineral grains and air, while saline water has high electrical conductivity. - Methods: Spontaneous, electrical resistivity, induced polarization, time and frequency domain electromagnetic methods, ground-penetrating radar, transient electromagnetic methods | - One-dimensional to four- dimensional electrical resistivity - Resistivity Depth - Resistivity Profiling - Electrical Resistivity Tomography - Very Low Frequency Electromagnetic profile | - Detect spatial or temporal variations in water content or salinity. - Covering large areas through land-based or airborne surveys, surpassing point sampling limitations. - Borehole logging methods complement survey data by providing vertical profiles of properties. - Monitoring snowmelt infiltration using time-lapse resistivity imaging, mapping water seepage with very low-frequency electromagnetic methods and characterizing active fractures in carbonate aquifers using a combination of techniques. | - Resolution of geophysical methods decreases significantly as depth increases within an aquifer, reducing their reliability in deeper areas. - The methods measure bulk resistivity, which differs from fluid resistivity. - It is necessary to have control points with direct measurements to validate the geophysical results obtained. - In resistivity profiles, there can be challenges in distinguishing between geological materials due to contrasts in resistivity. For example, the bedrock surface in coastal aquifers may resemble a freshwater layer, and a clay layer can mimic a saltwater layer in terms of resistivity. | - Buselli & Lu, 2001 - Nenna et al., 2013 - González et al., 2021 - Torrese & Pilla, 2021 - Alshehri & Abdelrahman, 2022 |
|  | Gravity surveying method | - Land-based gravity measurements are used to identify fluctuations in groundwater storage, indicating changes in underground water volume. - Gravity measurements are utilized to detect changes in groundwater storage through in situ observations and analysis of data from the gravity recovery and climate experiment satellite | - Gravity Recovery and Climate Experiment data (GRACE) - Gravity Recovery and Climate Experiment Follow-on data (GRACE-FO) - Other high-resolution satellite gravity datasets | - Improved precision of gravity meters enables accurate measurement of variations in gravitational attraction. - Gravimetric methods are now applicable in smaller study areas due to increased precision. - New-generation superconducting gravity meters allow for the collection of gravimetric data in small catchments. - Gravimeter recordings show noticeable influences from groundwater variations, soil water content, and snow coverage | - Estimating changes in groundwater storage using Land-based gravity measurements data involves significant uncertainties that arise from errors in Land-based gravity -derived total water storage changes and non-groundwater storage changes from models. - Inaccuracies in model-simulated storage changes, especially in soil moisture, contribute to uncertainties in groundwater storage estimates. - Surface water and glaciers, not considered in most land surface models, can introduce biases in groundwater storage estimates. - Errors are present in Land-based gravity -derived total water storage changes, requiring post-processing techniques to reduce noise, but this can lead to signal attenuation. - The spatial resolution of GRACE is limited to approximately 300 km or larger due to inherent measurement errors and satellite orbit configurations; poses limitations on its application in groundwater hydrology | - Feng, 2018 - Ikuemonisan & Ozebo, 2020 - Mohamed, 2020 - Bremard, 2022 - Ibrahim et al., 2023 |
|  | Remote sensing | Interferometric Synthetic Aperture Radar (InSAR)   - Measures ground displacement using radar interferometry, which captures shifts in the ground between different observations. - In order to use subsidence as an indicator of changes in groundwater storage, the aquifer needs to experience physical deformation caused by either groundwater depletion or recharge | - Sentinel-1 - ALOS-2 - Radarsat-2 - COSMO Skymed - TerraSAR-X/Tandem-X - GRACE - GRACE-FO | - The radar instrument is capable of making measurements even in the presence of cloud cover and without sunlight, and it has the ability to detect changes at a sub-centimeter level. - Global Navigational Satellite System measurements are valuable for obtaining long-term records of subsidence in different regions worldwide and for establishing connections between changes in groundwater storage from GRACE and surface deformation. - Altimetry allows for a broader spatial coverage and the provision of long-term subsidence records. - In areas where groundwater availability is known to be the primary factor driving subsidence, altimetry can be a useful tool for approximating groundwater conditions, especially when utilizing missions with extended repeat periods and smaller cross-track spacing. | - Changes in surface properties such as vegetation or soil moisture can reduce coherence and introduce noise. To overcome this, it is necessary to stack multiple observations over time to track the signal. - The successful application of InSAR for groundwater estimation depends on local geological conditions, surface cover, and factors such as atmospheric and ionospheric noise. Subsidence can be used as a proxy for changes in groundwater storage only if the aquifer undergoes physical deformation associated with groundwater depletion or recharge. - InSAR relies on the coherence of backscattered energy, and if surface features within a pixel change their relative position rather than undergoing a bulk shift within the pixel, interferometric measurements cannot be obtained. - Precipitable water vapor in the atmosphere can pose limitations, particularly in humid areas, as even small path delays of centimeters can introduce errors in the deformation signal. - The application and resolution of InSAR for groundwater problems are constrained by data availability, repeat coverage, and sampling. Currently, for global groundwater monitoring, only the Sentinel-1 mission by the European Space Agency provides adequate coverage and repeat coverage suitable for global assessments. | - Becker, 2006 - Antonio et al., 2019 - Farrag et al., 2019 - Bui et al., 2021 - Usman et al., 2021 - Adams et al., 2022 |
|  |  | Global Navigational Satellite System: Offers measurements at specific points |  |  |  |  |
|  |  | Radar altimetry: Ability to detect subsidence caused by groundwater and can indirectly measure changes in groundwater storage. |  |  |  |  |
|  |  | Lidar: Using land elevation change as an indicator to detect groundwater extraction |  |  |  |  |
|  | Machine Learning | - Machine learning techniques have been used in over-exploited basins to study groundwater storage change. - The applications of machine learning have been conducted at different spatial scales, including sub-regional and regional levels. - Deeper understanding of the complex and non-linear relationships among various factors that influence groundwater storage change. - Factors considered in these studies include agricultural water demands, surface water availability, groundwater supplies, and the type of water year. - Aid in improving understanding of the dynamics and drivers of groundwater storage change in these basins | - Hydrological data - Meteorological data - Methods (not limited to): - ANN (Ibrahem et al., 2022) - SVM (Support Vector Machine) (D. Liu et al., 2021) - ANFIS (Adaptive Neuro-fuzzy inference system) (Nourani et al., 2023) - Decision Tree (S. Hassan & Langroodi, 2015) | - Utilizing response surface regression provides a clear and definitive approach for addressing model uncertainty in groundwater level estimation. - ANN is implemented to forecast changes in groundwater storage (Ibrahem et al., 2022). - Response surface regression (RSR) is a computational tool that fits a polynomial regression model to estimate the response surface, enabling approximation of complex relationships between variables (Krishna et al., 2023). | - Prediction ability of models for groundwater fluctuations is based on network for monitoring and the systems for acquiring data. - Hybrid models are much accurate than stand-alone machine learning models | - Rahmati et al., 2019 - Liu et al., 2020 - J. Yin et al., 2021 - Ibrahem et al., 2022 - Krishna et al., 2023 |
|  | Hydrological models | SWAT (Soil & Water Assessment Tool): continuous-time simulation refers to a modeling approach that represents the dynamics of a system in a continuous manner, without discrete time intervals | - Hydrogeological data - Land use, soil characteristics, land elevation | - Exhibits versatility in terms of catchment size, making it suitable for both small and large catchments. - Incorporate the effects of land use changes. - Simulate sediment transport, vegetation growth, and nutrient transport, providing a comprehensive understanding of the system dynamics | - Dynamic land use is not taken into account, and the consideration of dew point is absent. - The complexity of the system necessitates human expertise and computational capacity for effective implementation | - Awan & Ismaeel, 2014 - Melaku & Wang, 2019 |
|  |  | MIKE11: simulation of water flow, water level, water quality, and sediment transport in various water bodies such as rivers, flood plains, irrigation canals, reservoirs, and other inland water systems |  |  |  | - H. L. Liu et al., 2007 - Dibaj et al., 2021 |
|  |  | WetSpass: Computes the cumulative actual evapotranspiration for each pixel by summing up the evaporation from open water, impervious surfaces, bare soil, interception of vegetated areas, and transpiration from the vegetation cover |  | Capability to simulate groundwater recharge even in regions where there is limited availability of observed data |  | - Giovanni & Donnell, 2001 - Armanuos et al., 2016 - Zdon et al., 2019 |
|  |  | Hydrus-1D: provides an environment for analysing the flow of water and the transport of solutes in porous media that are variably saturated |  | Highly effective in simulating the spatial and temporal variations in the ionic strength of soil | Requires a large amount of data for analysis and modelling | - (Leterme et al., 2012; Stafford et al., 2022) |
|  |  | Soil Water Balance model: Enable the estimation of potential groundwater recharge | Vegetation, soil characteristics, and meteorological data | Particularly focused on estimating the replenishment of groundwater | - Calculation of other components of the water balance is not included. - Requires a large amount of data for analysis and modeling | - Dripps & Bradbury, 2007 - Maihemuti et al., 2021 |
|  |  | MODFLOW: Modeling and forecasting the state of groundwater and the interactions between groundwater and surface water | Hydrogeological data | Simulation of both continuous and fluctuating groundwater movement in confined and unconfined aquifers can take into account the influence of wells, rivers, drains, boundaries dependent on hydraulic head, recharge, and evapotranspiration. | - Precise measurement of flow velocity is not achieved. - Conducting transient studies necessitates data to be collected within the same timeframe, which can be problematic | - Malekzadeh et al., 2019 - Zeydalinejad, 2022 |
